# Promising applications of D-amino acids in periprosthetic joint infection

**DOI:** 10.1038/s41413-023-00254-z

**Published:** 2023-03-10

**Authors:** Matthew Caldwell, Megan Hughes, Fei Wei, Christopher Ngo, Raven Pascua, Abinaya Sindu Pugazhendhi, Melanie J. Coathup

**Affiliations:** 1 Biionix Cluster & College of Medicine, University of Central Florida, 6900 Lake Nona Blvd, Orlando, FL 32827 USA; 2 School of Biosciences, Cardiff University, CF10 3AT Wales, UK; 3 Burnett School of Biomedical Sciences, College of Medicine, University of Central Florida, 6900 Lake Nona Blvd, Orlando, FL 32827 USA

**Keywords:** Pathogenesis, Bone

## Abstract

Due to the rise in our aging population, a disproportionate demand for total joint arthroplasty (TJA) in the elderly is forecast. Periprosthetic joint infection (PJI) represents one of the most challenging complications that can occur following TJA, and as the number of primary and revision TJAs continues to rise, an increasing PJI burden is projected. Despite advances in operating room sterility, antiseptic protocols, and surgical techniques, approaches to prevent and treat PJI remain difficult, primarily due to the formation of microbial biofilms. This difficulty motivates researchers to continue searching for an effective antimicrobial strategy. The dextrorotatory-isoforms of amino acids (D-AAs) are essential components of peptidoglycan within the bacterial cell wall, providing strength and structural integrity in a diverse range of species. Among many tasks, D-AAs regulate cell morphology, spore germination, and bacterial survival, evasion, subversion, and adhesion in the host immune system. When administered exogenously, accumulating data have demonstrated that D-AAs play a pivotal role against bacterial adhesion to abiotic surfaces and subsequent biofilm formation; furthermore, D-AAs have substantial efficacy in promoting biofilm disassembly. This presents D-AAs as promising and novel targets for future therapeutic approaches. Despite their emerging antibacterial efficacy, their role in disrupting PJI biofilm formation, the disassembly of established TJA biofilm, and the host bone tissue response remains largely unexplored. This review aims to examine the role of D-AAs in the context of TJAs. Data to date suggest that D-AA bioengineering may serve as a promising future strategy in the prevention and treatment of PJI.

## Introduction

When combined, physicians in Australasia, the United Kingdom, and North America together perform ~1.5 million primary total joint arthroplasties (TJAs) annually.^1–4^ Due to significant improvements in pain, function, and quality of life, TJAs are considered among the most successful orthopedic procedures,^1^ and their use is increasing. In 2010, 719 000 total knee arthroplasties (TKAs) and 332 000 total hip arthroplasties (THAs) were performed in the United States, and by 2030, this number is projected to grow by 673% to 3.48 million TKA procedures and 174% to 572 000 THA procedures.^5,6^ The growth rates of upper extremity arthroplasty are comparable.^7^ For example, by 2030, the demand for primary shoulder arthroplasties among younger patients (≤55 years of age) is projected to increase by 333.3% and by 755.4% in patients older than 55 years of age.^8^ As such, the commonness of TJA procedures is increasing at an accelerated rate, with a total of 3.8 million annual surgeries expected to be performed in 2030.^6,7,9^ Further consideration is the need for future revision arthroplasties. Similar gains are expected for revision THA and TKA procedures, which are expected to grow by 142% (72 000 procedures) and 190% (120 000 procedures), respectively, by 2030.^10^ Revision THA is expected to reach 110 000 procedures (a 219% increase), and revision TKA is expected to reach 253 000 procedures (a 400% increase) annually by 2060.^11^ Although the demand for TJAs across all age groups and in males and females is increasing,^8,12,13^ a disproportionately greater affect is anticipated in elderly patients (≥65 years) to support mobility in older age. This will undoubtedly have substantial future economic implications due to our aging population, which is rapidly progressing toward a super-aging society where 20% of the population is projected to be aged ≥65 years by the year 2050.^14–16^ An increase in our oldest population (aged >85 years) and an increase in people living to old age than ever before will undoubtedly increase the need for TJAs. The implications for this include the direct healthcare costs of increased primary and revision surgery, the indirect societal burden of missed productivity owing to time away from work, and the increased need for qualified surgeons to meet the demand.^8^

**Fig. 1:**
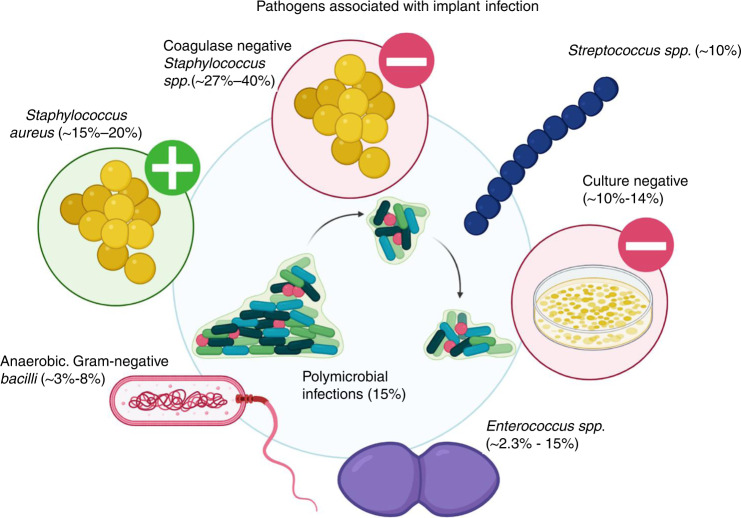
A schematic presenting examples of the various pathogens associated with orthopedic PJIs and their estimated incidence.^34,36,37^ Mono- and polymicrobial infections are associated with PJI, with up to 15% of all cases being comprised of multiple bacterial species (*spp*.). *Staphylococcus and* coagulase-negative *staphylococci* are involved in 50%–60% of PJIs

Periprosthetic joint infection (PJI) is one of the leading causes of TKA and THA failure^17–19^ and is one of the most common reasons for revision shoulder and elbow arthroplasty.^20^ The incidence of PJI is estimated to range between 0.7% and 5% in elective cases involving TJA and upward of 30% in complex trauma cases.^21–28^ Despite the consistently low incidence of PJI, the rising number of TJAs means that the overall burden of PJI is also rapidly increasing,^1^ and the rate more than doubled between 2001 and 2011.^29^ Gram-positive organisms are associated with the majority of PJIs and are also implicated in 70%–80% of polymicrobial infections.^1^ The most common etiological agents are coagulase-negative *Staphylococci* (~27%–40%), *Staphylococcus aureus* (*S. aureus*, ~15%–20%), *Streptococci* (~10%), *Enterococci* (*~*2.3%–15%*)*, other gram-negative strains including *Escherichia coli, Pseudomonas aeruginosa, Klebsiella pneumoniae*, and *Acinetobacter baumannii* (together ~5%), and anaerobes (~3%–8%) (Fig. 1).^30–34^ ‘Culture negative’ PJI, where clinical indications of infection are present but no microorganisms can be isolated,^35^ ranges from a 5%–41% rate, with 10%–14% being the generally accepted estimate.^34,36,37^ The emergence of antimicrobial resistance among these species has further complicated the treatment of PJI. A prominent example is methicillin-resistant *S. aureu*s (MRSA), which in the wider medical context resulted in >100 000 deaths globally,^23^ with overall bacterial antimicrobial resistance reported to be associated with ~4.95 million deaths in 2019.^38^

**Fig. 2:**
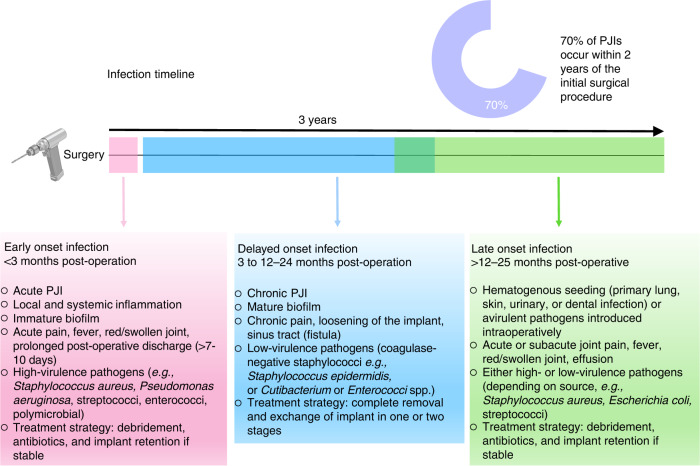
*S. aureus* and aerobic gram-negative bacilli together contribute to ~60% of early-onset (<3 months) infections.^34^ Polymicrobial infections are also higher at this time. Typically, delayed-onset PJI (3 months to 12–24 months postsurgery) occurs at the time of surgery and due to inoculation with less virulent microorganisms. At this stage, coagulase-negative staphylococci and enterococci are more common. Late-onset PJI (>12 to 24 months postsurgery) occurs mostly following hematogenous seeding from a primary infection located elsewhere in the body; *S. aureus* predominates in this situation. Late-onset PJI is less common and is often due to inoculation with relatively avirulent microorganisms peri-surgically

Several classification schemes exist, and PJI is typically classified as early, delayed, or late onset (Fig. 2).^34,39,40^ Generally, early-onset infections occur <3 months following the last procedure and *via* infections initiated at the time of surgery by relatively virulent microorganisms (e.g., *S. aureus*, streptococci, enterococci).^34,41^ Delayed-onset PJI occurs after 3 months but before 12 or 24 months and is considered to be acquired at the time of surgery but is caused by less virulent microorganisms (e.g., coagulase-negative *Staphylococci*).^42^ Late-onset PJIs occur >12 to 25 months after surgery and are frequently due to hematogenous infection (*S. aureus* is reported in up to 34% of cases^43^) but may also be caused by indolent infection caused through intraoperative inoculation. However, the majority (65%) of PJIs occur within 1 year of surgery.^34,44^ When PJIs develop, quality of life and function are severely decreased,^42,45–47^ health care costs increase up to 5-fold^46,48^ (US$30 000–120 000 per patient,^49–52^) and surgical amputation of the affected limb may be performed to resolve the complication.^53^ Furthermore, PJIs are associated with mortality rates of 2%–4% within 90 days^54,55^ and 20%–26% within 5 years^54,56^ postinfection; the 5-year mortality rate is reported to be greater than that of four of the five most commonly diagnosed cancers in the United States.^1,57^ Challenges remain in the successful treatment of PJIs. Corrective procedures often involve a 1- or 2-stage revision surgery, with successful outcomes reported to be 55%–88% when treating staphylococcal infections,^31,58^ 69% when treating pseudomonal PJIs,^59^ and a 5%–25% risk of reinfection that escalates as the number of revision surgeries increases.^60^ As a result, in the case of multiple revisions, the success rate of joint reconstruction and subsequent limb retention is reduced to 43%–62%.^61^

A major challenge in the successful treatment of PJI is the persistence of microorganisms on the implant surface in the form of biofilms. Planktonic bacteria are able to compete, adhere, and colonize a surface, and infections form due to bacterial adherence and subsequent biofilm formation.^62^ Biofilms are a complex, functional, self-produced multilayered exopolymeric matrix consisting of polysaccharides, proteins, extracellular DNA (eDNA) released by bacterial autolysis, and lipids that surround bacterial communities as protective barriers.^63^ As such, microorganisms are shielded from environmental stressors, including antibiotics and immune responses, making them difficult to treat and eradicate.^64^ Additionally, the release of cell-to-cell signaling molecules and chemical cues (quorum sensing) induces bacteria within a population to respond in concert by changing patterns of gene expression that lead to biofilm differentiation.^65^ Furthermore, biofilms adhere to surfaces, including the common stainless steel and titanium alloy metal components used in orthopedic devices.^66^

Over the past two decades, only two new classes of antibiotics have been approved for medical use, namely, oxazolidinones and cyclic lipopeptides.^67^ Given the current challenges to antibiotic development and research, a novel approach is urgently needed to directly address the issue of treating infections, including orthopedic-related infections. Bacteria are able to regulate biotic and abiotic surface adhesion, biofilm formation, maturation, and dispersal in multiple ways, but one of growing interest is the use of D-amino acids (D-AAs) (Table 1). D-amino acids are classified as antimicrobial peptides (AMPs), which are a diverse group of naturally occurring small-sized peptides consisting of a large number of lysine or arginine residues. The production of various D-AAs by bacteria is essential for their adaptation to diverse environmental threats. Further elucidation of D-AA synthesis, metabolism, function, and activity will undoubtedly contribute to our understanding of the bacterial strategies used for environmental evasion.^68^ Although an area of significant medical and clinical interest, our scientific knowledge on the use of D-AAs within the orthopedic setting is limited, and much remains to be revealed. As such, the aim of this review focuses on exploring and assessing the efficacy of D-AAs in the context of PJI and determining whether they may offer the future promise of a novel preventative and therapeutic approach to inhibit the adhesion, formation, and maturation of pathogenic biofilms, as well as their dispersal.

**Table 1:** A table presenting the chemical structure, molecular weight, chemical formula, and chemical name of the D-AAs

D-Amino Acid	Chemical Structure	Molecular Weight	Molecular Formula	Chemical Name
D-Alanine		89.09	C_3_H_7_NO_2_	(2*R*)-2-aminopropanoic acid
D-Arginine		174.20	C_6_H_14_N_4_O_2_	(2*R*)-2-amino-5-(diaminomethylideneamino)pentanoic acid
D-Asparagine		132.12	C_4_H_8_N_2_O_3_	(2*R*)-2,4-diamino-4-oxobutanoic acid
D-Aspartic acid		133.10	C_4_H_7_NO_4_	(2*R*)-2-aminobutanedioic acid
D-Cysteine		121.16	C_3_H_7_NO_2_S	(2*S*)-2-amino-3-sulfanylpropanoic acid
D-Glutamic acid		147.13	C_5_H_9_NO_4_	(2*R*)-2-aminopentanedioic acid
D-Glutamine		146.14	C_5_H_10_N_2_O_3_	(2*R*)-2,5-diamino-5-oxopentoic acid
D-Histidine		155.15	C_6_H_9_N_3_O_2_	(2*R*)-2-Amino-3-(1*H*-imidazole-4-yl)propanoic acid
D-Isoleucine		131.17	C_6_H_13_NO_2_	(2*R*,3 *R*)-2-amino-3-methylpentanoic acid
D-Leucine		131.17	C_6_H_13_NO_2_	(2*R*)-2-amino-4-methylpentanoic acid
D-Lysine		146.17	C_6_H_14_N_2_O_2_	(2*R*)-2,6-Diaminohexanoic acid
D-Methionine		149.21	C_5_H_11_NO_2_S	(2*R*)-2-amino-4-methylsulfanylbutanoic acid
D-Phenylalanine		165.19	C_9_H_11_NO_2_	(2*R*)-2-amino-3-phenylpropanoic acid
D-Proline		115.13	C_5_H_9_NO_2_	(2*R*)-pyrrolidine-2-carboxylic acid
D-Serine		105.09	C_3_H_7_NO_3_	(2*R*)-2-amino-3-hydroxypropanoic acid
D-Threonine		119.12	C_4_H_9_NO_3_	(2*R*,3*S*)-2-amino-3-hydroxybutanoic acid
D-Tryptophan		204.22	C_11_H_12_N_2_O_2_	(2*R*)-2-amino-3-(1*H*-indol-3-yl)propanoic acid
D-Tyrosine		181.19	C_9_H_11_NO_3_	(2*R*)-2-amino-3-(4-hydroxyphenyl)propanoic acid
D-Valine		117.15	C_5_H_11_NO_2_	(2*R*)-2-amino-3-methylbutanoic acid

Ball and Stick components: red = oxygen, blue = nitrogen, yellow = sulphur, and white = hydrogen. Information was sourced from the National Library of Medicine, National Center for Biotechnology information https://pubchem.ncbi.nlm.nih.gov

## A brief overview of AMPs

Bacteria are ubiquitous and diverse single-celled organisms that are noted for their commensal or pathogenic properties. To gain dominance, all bacteria naturally produce antimicrobial bioactive compounds and biomolecules (e.g., peptides,^69,70^ carbohydrate pradimicins,^71^ anionic biosurfactants,^72^) which are either lethal (bactericidal) or inhibitory (bacteriostatic) to other bacteria and biofilms, as well as to viruses and fungi.^73,74^ The primary difference between a commensal and pathogen is those commensals do not encode such aggressive tools for invasion, and the host’s strategy is more or less to ignore them.^75^ Although commensals can be harmful, these bacteria are more often beneficial to humans. For example, lactobacilli act as probiotics to the human gut mucosa through the secretion of substances including AMP bacteriocins, which include lantibiotics that are ubiquitously produced by gram-positive lactobacilli (e.g., nisin A,^76^ pediocin PO2, lacticin 3147, BH5, JW3, and NK24,^77,78^) non-lantibiotics produced by gram-negative bacteria (e.g., garvicin Q, microcins, colicins, pyocins, tailocins,^78,79^) organic acids (e.g., valeric, propionic, acetic, formic, lactic, caproic, and butyric acids,^80,81^) and hydrogen peroxide. The commensals compete for nutrients and space and use pivotal mechanisms against the pathogens, including membrane permeabilization^82,83^ with interference of the proton motive force,^84^ essential enzyme and subsequent protein synthesis, gene expression, and upregulation of the host’s immune system.^82^ The secreted compounds induce cell membrane pore formation, the efflux of ions, and changes in membrane potential that eventually inhibit pathogenic bacterial growth and/or cause death.^85–88^ As such, these compounds contribute to preventing the adhesion, proliferation, and viability of pathogens that cause disease.^89,90^ As such, AMPs are considered highly promising because they display broad spectrum activity, a low propensity to induce resistance, and high effectiveness at low concentrations;^73,91^ AMPs have shown antibacterial, antifungal, and antiviral activity, as well as immunomodulatory activity.^92^

Generally, the biodiversity of compounds found in bacteria means that they are considered an untapped reservoir for promising biomolecules with varying structural and functional antimicrobial activity. Although some AMPs are anionic,^93^ their positive charge enables interaction with the bacterial membrane, which is largely negatively charged.^73^ Extensive studies on the structure-activity relationship have revealed that net charge, hydrophobicity, and amphipathicity are together the most important physicochemical and structural determinants providing AMPs with antimicrobial potency and cell selectivity.^92^ Studies have shown excellent antibacterial activity *via* multiple targets on the plasma membrane and intracellular targeting.^91^ For example, the cyclic peptides mathiapeptide A, destotamide B, marfomycins A, B, and E, spirotetronate polyketides abyssomycin C and lobophorin F and H, and alkaloid and sesquiterpene derivatives caboxamyxin and mafuraquinocins A and D have each been isolated from various bacterial species.^73^ These peptides are reported to possess antimicrobial properties that have the ability to eradicate *S. aureus*, MRSA, *Micrococcus luteus*, *Bacillus subtilis*, and *Enterococcus faecalis.*^73,94^ Pradimicins A, B, and C present broad-spectrum anti-fungal activity with efficacy against *Candida spp*., *Cryptococcus neoformans*, *Aspergillus spp*., dematiaceous molds, and Zygomycetes.^95^ Furthermore, iturin, lichenycin, and fengycinic lipopeptides produced by *Bacillus subtilis* also possess strong and broad-spectrum antifungal activity, including against *Pseudomonas spp*.^96^ and *Colletotrichum acutatum,*^97^ by creating pores that destroy the fungal cell membrane.^95,98,99^ *P. aeruginosa* makes three known antifungal compounds, namely, dihydroaeruginoic acid,^100^ pyocyanin, and 1-hydroxyphenazone.^101^ Given the continuing emergence of multidrug-resistant pathogens and the absence of novel antibiotics, the potential role of these peptide toxins to act as an antimicrobial therapeutic option in humans, either independently or as a combination therapy, is gradually being uncovered and is an area where further discovery and scientific understanding are critically needed. Several AMPs (e.g., polymyxin, vancomycin, and daptomycin) have already been approved for human use, and other natural and synthetically designed AMPs are in various stages of clinical development.^102,103^ The production of new synthetic antimicrobials through chemical and structural modification of natural products, as well as the biomanufacturing of natural antimicrobials, is an area of high significance that may play a major role in resolving orthopedic PJIs and other biotic and abiotic infections.

## D-AAs: production and role

Although more than six decades ago, high concentrations of exogenous D-AAs were shown to inhibit bacterial growth,^104,105^ the emerging efficacy of D-AAs, their rich abundance and diversity, and their roles in microbial physiology, modulation of the cell-wall structure, and the dissolution of biofilms^106^ are just beginning to be appreciated.

### Prokaryote production of D-AAs

All protein-forming amino acids, with the exception of Gly, consist of one chiral α-carbon and are therefore able to exist in the following two stereoisomeric forms: the levorotatory (L) and the dextrorotatory (D) forms.^107^ D-amino acids are thus isomers of L-amino acids, making them nonsuperimposable mirror images of one another. L-amino acids are substantially more abundant and essential for life, as they provide the building blocks for ribosomally produced polypeptides and are key metabolic intermediaries in biological systems.^108,109^ Amino acid racemases are enzymes that catalyze reversible stereo-chemical interconversion (e.g., alanine (Ala) racemase (EC 5.1.1.1), glutamate (Glu) racemase (EC 5.1.1.3), aspartate (Asp) racemase (EC 5.1.1.13), serine (Ser) racemase (EC 5.1.1.18), proline (Pro) racemase (EC5.1.1.4), lysine (Lys) racemase (EC 5.1.1.5), arginine (Arg) racemase (EC 5.1.1.9), and histidine (His) racemase (EC 5.1.1.24),^68,106,107,110^ enabling bacterial production of D-AAs through racemization of both proteinogenic and nonproteinogenic L-amino acids. This occurs in either a pyridoxal-5-phosphate (PLP)-dependent or -independent manner; thus, L-amino acids typically act as the substrate for the generation of D-AAs.^111^ The mechanisms involved are complex, and previous review articles have comprehensively covered this topic.^107,112^ In brief, the PLP-dependent mechanism typically creates, e.g., D-Ala, D-Ser, and D-Arg, through two trans-aldimination reactions, while PLP-independent mechanisms are more varied and generate, *e.g*., D-Pro, D-Asp, and D-Glu.^107,112^ It is now accepted that diverse bacterial species produce and release different types of D-AAs into the environment in a millimolar range when cultured.^109,113^ Recent works have begun to highlight both the abundance and potential role of D-AAs in nature; however, the synthesis enzymes reported to date cannot account for the diversity of D-AAs identified in bacteria or within bacterial-rich environments. As described above, D-AAs can be synthesized by highly specific enzymes; however, broad-spectrum amino acid racemases have also been identified in some bacteria.^107,108^ Furthermore, various bacteria are also known to produce specific molecular signals resulting in the generation of free-branched D-AAs, such as D-isoleucine, D-leucine, and D-valine, which are synthesized via epimerization (e.g., ile 2-epimerase^110^) and not racemization.^114,115^ Finally, a further class of D-AAs are synthesized via reversible stereospecific amination of α-keto acids, catalyzed by alternative PLP-dependent enzymes, including aminotransferase enzymes.^109,116^ The biological roles of these diverse and abundant D-AAs remain largely unknown.

### Role of D-AAs in prokaryotes

D-amino acids have been reported to be associated with bacterial adhesion, growth, biofilm formation and dispersal, and the regulation of peptidoglycan metabolism, where disruption of their synthesis leads to cell death.^108,109,117^ Some D-AAs are inherently bioactive, whereas others are building blocks for important biomolecules such as lipid II, the bacterial cell wall precursor.^115^ Increased D-AA concentrations may indicate to the bacteria that nutrients are limited and dispersal to a planktonic state is favored,^112,118^ and it has been suggested that D-Phe and D-Leu may be used among bacteria to outcompete other species via biofilm inhibition.^108^ Therefore, the enzymes responsible for D-AA synthesis are also promising targets for antibacterial therapeutics. The major sources of D-AAs in prokaryotes are extracytoplasmic in gram-positive organisms or periplasmic polymeric biomolecules in gram-negative species, including peptidoglycan, teichoic acids, and poly-γ-glutamate. Peptidoglycan is the major component of the bacterial cell wall and the most commonly cited source of D-AAs in bacteria.^109^

#### Role of D-AAs in the prokaryotic cell wall

Bacteria have a robust and multitasked ability to withstand many physical, chemical, and biological insults. A major component of this is due to the plasticity of the peptidoglycan cell wall matrix, which fortifies the cytoplasmic membrane supporting the cell in terms of shape, strength, and subsequent resistance to osmotic pressure.^119–121^ The critical requirement of peptidoglycan for bacterial propagation, together with its potential value as an antibiotic target, has led to renewed interest in the study of peptidoglycan synthesis and function.^122^ However, much remains to be elucidated. Peptidoglycan is found on the external surface of the cytoplasmic membrane of almost all bacteria, serves as a scaffold for anchoring other-cell-envelope components^123^ and is essential for cell viability.^112^ Peptidoglycan consists of a basic unit made of the disaccharide *N*-acetyl-glucosamine-*N*-acetyl-muramic acid, and, most notably, the incorporation of D-Glu and D-Ala are key components of peptidoglycan.^68,121^ The incorporation of these D-AAs into the peptidoglycan structure provides protection and cell wall resistance to most proteases that target and cleave L-amino acids. D-Ser and D-Asp are often present in the terminal position of the stem peptide and provide tolerance to certain bactericidal agents, including vancomycin.^108,112,124–127^ As such, in the stationary phase, D-AAs control peptidoglycan chemistry, density, remodeling, and strength in D-AA-producing and nonproducing bacteria.^113,128^ The impairment of D-AA production leads to excessive accumulation of peptidoglycan and hypersensitivity to osmotic shock. Thus, the presence of D-AAs likely constitutes a bacterial adaptation to protect a vital cellular structure. Interestingly, this structural role can be therapeutically exploited, with the application of D-Ser in combination with beta-lactam antibiotics having a synergistic effect against MRSA by substituting the D-Ala-D-Ala bonding for D-Ala-D-Ser, thus impairing transpeptidation.^129^

#### Role of D-AAs in prokaryotic spore germination

The majority of bacterial species commonly associated with PJIs are non-spore forming. However, spore-forming aerobes, including *Bacillus spp*. and anaerobes such as *Clostridium spp*.^130,131^ account for ~3%–8% of orthopedic implant-associated infections.^30–34^ Bacterial spores are robust, vegetative, and metabolically dormant, and are produced to survive the severe and adverse climatic conditions of starvation and stress.^132,133^ Exposure to germinants induces the germination of spores, which are highly resistant to varying ranges of temperature, pressure, desiccation, ultraviolet radiation, pH extremes, and noxious chemicals, including hypochlorite, aldehydes, ethylene oxide, and several other extreme conditions.^132,134^ In the presence of specific small molecule germinants such as L-Ala or other nutrients, spores interact via germinant-specific receptors to reactivate their metabolism and allow for vegetative growth in *Bacillus spp*.^135^ Remarkably, D-Ala displayed anti-germinant properties against bacterial spores by preventing premature germination. Chesnokova and colleagues^136^ demonstrated that the enzyme alanine racemase (Alr) is present within the basal layer of spores and is capable of converting the spore germinant L-Ala to the germination inhibitor D-Ala. The authors suggested that an important function of Alr is to produce D-Ala during the late stages of sporulation to suppress germination of the developing spore, presumably as a mechanism to prevent premature germination when under low nutrient or adverse environmental conditions. McKevitt et al.^137^ reported that D-Ala may also alter the kinetics of germination in vivo and enhance the temporal efficacy of infection. Furthermore, D-His has also been implicated as a germination inhibitor following infection in murine macrophages; however, its mechanism remains elusive.^138^

#### Role of D-AAs in prokaryotic metal scavenging and immune host cell evasion

Beyond supporting the integrity of the bacterial cell wall and the regulation of spore germination, D-AAs also contribute to metal scavenging and host cell evasion. Metal procurement is a critical microbial process in metal-deficient conditions, such as inside an infected host. To regulate and limit pathogenesis, humans and other mammals rapidly and dramatically restrict access to essential metals in a process termed “nutritional immunity”. This extends to many micronutrients, including iron, nickel, cobalt, copper, manganese, and zinc.^139,140^ However, invading bacterial pathogens have developed numerous and varied adaptive strategies to circumvent nutritional immunity. These mechanisms involve the use of countermeasures able to improve metal uptake, thereby facilitating survival, as comprehensively described in previous reviews.^141,142^ In 2016, Gheesin et al.^143^ discovered staphylopine, a novel metal-scavenging molecule produced by *S. aureus*. Staphylopine is synthesized *via* a combination of D-His, amino butyrate, and pyruvate prior to release into the extracellular environment, where it traps target metals in the affinity order zinc, cobalt, copper, and iron. Using a metal import system, the bacteria are subsequently able to recover these target metals, avoiding the metal starvation state imposed by the host. Furthermore, the study showed that staphylopine-deficient *S. aureus* exhibited reduced virulence during host infection. Interestingly, Anfora et al.^144^ demonstrated that accumulated D-Ser acts as a signal for hypercolonization and virulence gene expression in a murine model of *E. coli* infection. Similar results have been reported more recently.^145,146^ The mechanism remains elusive; however, D-Ser may be involved in cell growth and/or incorporation into peptidoglycan, where alterations in cell wall structure may subsequently alter virulence.

Found in chemosensory cells within the upper respiratory epithelium when stimulated, sweet taste receptor (T1R) inhibits the release of AMPs by neighboring cells. Bitter taste receptors (T2Rs) detect damaging molecules, including secreted bacterial products, and stimulate surrounding cells to release AMPs.^147^ AMPs, together with other secreted factors, contribute to mucosal innate immunity and the maintenance of a clean airway.^148^ Lee et al.^149^ demonstrated that *Staphylococcus spp*. in the nasal cavities of chronic rhinosinusitis patients produced D-Leu and D-phenylalanine (D-Phe), both of which can activate T1R. A more recent study by Lee et al.^147^ confirmed in vitro that D-Phe and D-Leu inhibited the release of AMPs and increased host cell death in response to infection with MRSA. The results also showed that these D-AAs inhibited beneficial T2R-mediated signaling, as well as the formation of *P. aeruginosa* biofilms, suggesting that D-AAs can inhibit innate immune responses through T1R and T2Rs and may play a major role in pathogenesis within the airway. Furthermore, a study by Kepert et al.^150^ demonstrated that D-tryptophan (D-Trp) produced by probiotic strains acted as an immunomodulatory substance by decreasing the production of TH2 cytokines and chemokines in human peripheral and murine immune cells while also ameliorating allergic airway inflammation when given to mice.

Although these studies were not focused on application in the orthopedic setting, of interest in this review are the bacterial species described. *S. aureus*, *E. coli*, *P. aeruginosa*, and *Staphylococcus spp*. are all highly relevant to PJIs, and together, these studies highlight a prominent role of D-AAs in facilitating bacterial survival in the host and their involvement in inhibiting the innate immune response, both key to the initiation and progression of PJI. Given this information, targeted investigation of D-AAs in PJIs, such as in osteomyelitis, appears highly warranted. Studies in this area may uncover new scientific knowledge that would aid in understanding bacterial evasion and survival strategies on implant surfaces, as well as within the confined structures of canaliculi or lacunae where bacteria are often protected from immune cell attack and can survive for long periods of time. The extent of bacterial invasion within this nano- and microporous network remains unknown and may be a primary factor in the development of chronic osteomyelitis. However, the role of D-AAs, if any, remains uninvestigated.

## Bacterial adhesion, biofilm formation, and dispersal at the orthopedic implant surface; the potential of D-AAs as countermeasure agents

### Inhibition of bacterial adhesion

Following surgical insertion, expeditious integration of the implant surface with host cells is critical for preventing competitive bacterial adhesion and subsequent colonization, also known as “the race to the surface”. The bacterial surface is highly organized, and one of its major functions is to facilitate adhesion, although the molecular and physical interactions that determine adhesion to biomaterials are not fully understood. Initial adhesion can be instantaneous, unspecific and reversible, occurring via but not limited to van der Waals forces, gravitational forces, surface electrostatic charge, hydrophobic interactions, Lewis acid-base interactions, and hydrogen bonding.^63^ Secondary to these adhesive forces, molecular-specific reactions occur between bacterial adhesins and through surface polymeric filamentous cell appendages, including pili, capsules, fimbriae, and pilus-like adhesive structures.^65,151,152^ Following this, in bacterial species adept at secreting an extracellular polysaccharide (EPS) matrix, irreversible attachment is facilitated, and biofilms are formed if furnished with a suitable supply of nutrients. Environmental factors affect initial bacterial adhesion, including fluid flow, temperature, exposure time, bacterial concentration, chemical treatment, and the presence of antibiotics.^65,152,153^ Furthermore, surface properties (e.g., surface chemistry, porosity, roughness, surface energy) are also considered major factors that influence bacterial adhesion.^154^ Specifically, implant surface roughness at the nanoscale has been reported to regulate the degree of van der Waals forces, thereby promoting adhesion. This phenomenon was reported with both titanium and nano-phased alumina,^155–159^ where surfaces with nanophase ZnO and TiO_2_ had less adhesion.^160^ Bacterial-related factors that affect adhesion include gram-positive or gram-negative surface energy and charge, outer membrane molecular receptor expression, and hydrophobicity.^151,152,161^ The properties of each of these implant and bacterial factors are interconnected, thus introducing much complexity. While this review has summarized the implant surface-related factors of bacterial cell adhesion, it is important to note that bacteria also adhere to host proteins, including the extracellular matrix molecules fibronectin, fibrinogen, and laminin,^162,163^ and adhere to and invade host cells, thereby evading host defenses and contributing to the pathogenesis of osteomyelitis.^164–168^

**Fig. 3:**
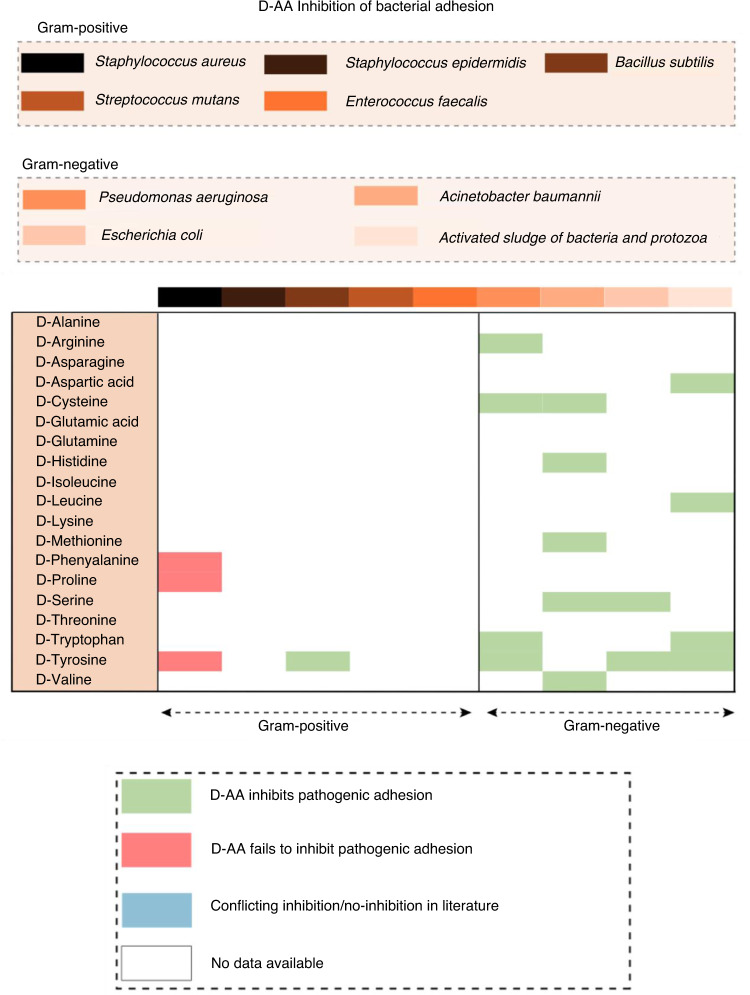
A comparative heatmap of data compiled from the literature showing the various gram-positive and gram-negative microorganisms associated with PJI and the efficacy of D-AAs to inhibit pathogenic bacterial adhesion to an abiotic surface. Based on the D-AAs examined thus far, studies suggest a beneficial role. There has been a dominant focus on exploring the effects of D-AAs on glass or polypropylene surfaces. Future studies that examine the response of pathogenic bacteria to D-AAs when exposed to orthopedic-related materials, e.g., medical grade titanium alloy, ceramics, polyetheretherketone, and highly crosslinked polyethylene, are warranted. Furthermore, to date, few studies have investigated the effect of D-AAs on many of the gram-positive species associated with PJI

Novel approaches able to prevent or reduce early bacterial adhesion to an implant surface, thereby favoring host cell adhesion at the expense of bacterial adhesion, may be a crucial step in preventing PJI (Fig. 3). A study by Hochbaum et al.^169^ reported that the D-AAs D-Pro, D-Phe, and D-Tyr did not prevent the initial attachment of *S. aureus* cells onto a glass or epoxy surface but blocked the subsequent growth of the foci into larger assemblies of cells, thus preventing biofilm formation. However, a more recent study demonstrated that exogenous D-Tyr significantly inhibited *E. coli* adhesion to a surface.^170^ Interestingly, using surface thermodynamic theory, this study reported that the total surface interaction energy increased when more D-Tyr was present, that the contribution of Lewis acid–base interactions relative to the change in the total interaction energy were much greater than the overall nonspecific interactions; furthermore, analysis of atomic force microscopy data suggested that the hydrogen bond numbers and adhesion forces decreased with increasing D-Tyr concentrations. In summary, D-Tyr contributed to the repulsion between the cell and the surface and ultimately led to the inhibition of bacterial adhesion. Similarly, Yu et al.^171^ demonstrated significant D-Tyr efficacy against *E. coli*, *P. aeruginosa*, and *B. subtilis* adhesion to a glass surface when studied under flow conditions. The effect was greatest against *B. subtilis;* however, the mechanism of action remains elusive and is considered unrelated to bacterial hydrophobicity or surface charge. Su et al.,^172^ using an “activated sludge” containing many kinds of bacteria and protozoa, demonstrated that D-Tyr, D-Asp, D-Trp, and D-Leu independently led to a significant reduction in cell adhesion efficiency while also increasing desorption efficiency to a flat membrane surface composed of polyvinylidene fluoride. Xu and Liu^173^ also reported the significant surface inhibitory effects of D-Tyr on activated sludge adhesion when investigated on glass and polypropylene slides. In this study, the administration of D-Tyr significantly inhibited the synthesis of autoinducer-2, eDNA and extracellular polysaccharides and proteins. Rumbo et al.^174^ evaluated the activity of 18 D-AAs on the pathogens *A. baumannii* and *P. aeruginosa* and demonstrated that pathogenic attachment to human alveolar cells was significantly reduced in *A. baumannii* by D-Cys, D-His, D-Met, D-Val and D-Ser in vitro. Additionally, the death of alveolar cells infected with *P. aeruginosa* was significantly reduced by D-Cys, D-Trp, and D-Arg; thus, these D-AAs showed an important protective effect against infection of these cells. Similarly, Connolly et al.^145^ reported that the addition of 1 mmol·L^−1^ D-Ser reduced the attachment of *E. coli* to HeLa cells. The mechanism(s) remains undiscovered, but at concentrations beyond the physiological level of production, D-Tyr has been shown to replace D-Ala in the peptidoglycan layer, affecting its thickness, the anchorage of surface proteins and hence cell‒cell or cell-surface interactions.^113,175^ Taken together, the results of these studies are highly encouraging, and D-AAs may provide a potential agent for the control of microbial orthopedic implant surface and host cell adhesion. To this end, and to the best of our knowledge, no studies have investigated the effect of D-AAs on bacterial adhesion to metal, ceramic, highly crosslinked polyethylene surfaces, or other materials within the context of orthopedic applications. As such, further investigation and discovery in this area may introduce a significant beneficial strategy in the context of PJI.

### Biofilm growth, maturation, and dispersal

Bacteria exist in a planktonic and, following adhesion to a biotic or abiotic surface, a biofilm state. Biofilms are complex and structured bacterial communities enclosed within a self-produced EPS matrix typically composed of protein, exopolysaccharide, and eDNA that are able to adhere to orthopedic implant surfaces.^162^ Typically, biofilm formation occurs in several stages, including bacterial adherence, the formation of microcolonies, the development of young biofilm, differentiation of structured mature biofilm, and the dispersal of mature biofilm.^162,176,177^ The formation of biofilms is the main pathogenic mechanism that leads to the chronicity and irreducibility of PJIs, making them a serious health care issue.^63,162^ Specifically, the presence of biofilms augments bacterial resistance against routine antibiotics by ~1 000-fold,^177^ and biofilms are resistant to desiccation, environmental stress, and exposure to UV light.^178^ As such, interrupting biofilm formation or preventing biofilm dispersal is an appealing strategy to combat PJIs and prevent their chronic development.

#### Gram-positive organisms in PJIs and the effect of D-AAs

##### Staphylococcus aureus

**Fig. 4:**
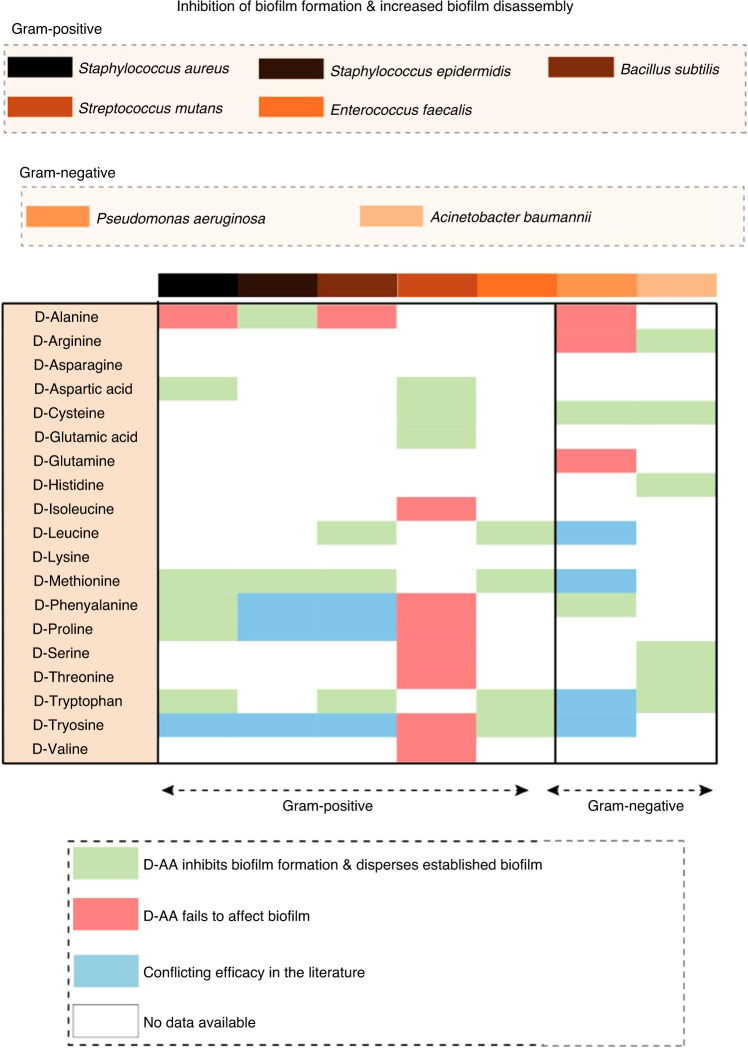
A comparative heatmap of data compiled from the literature showing the various gram-positive and gram-negative microorganisms associated with PJI and the efficacy of D-AAs to inhibit pathogenic biofilm formation and/or augment the disassembly of mature biofilms. Variability in the response to D-AAs for both gram-positive and gram-negative species was found. For example, all studies reported thus far have shown D-AA efficacy against *A. baumannii* biofilms; however, many of the D-AAs investigated against *S. mutans* were reported to be ineffective

**Fig. 5:**
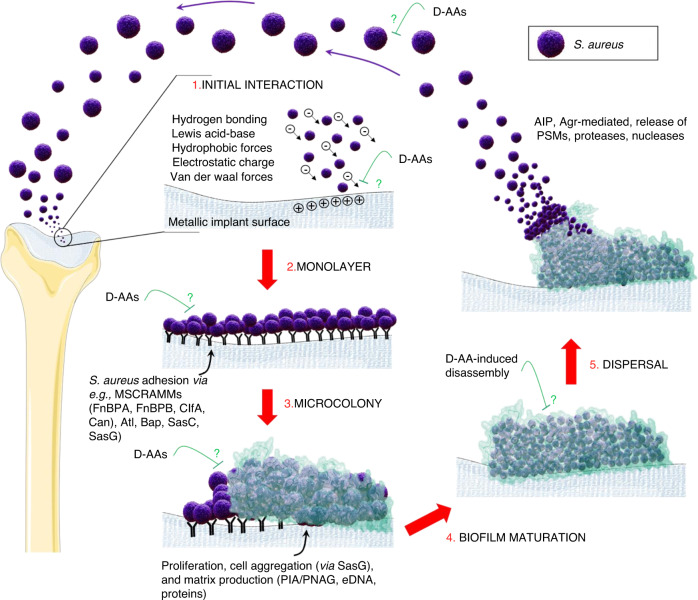
An example schematic showing the life cycle of *S. aureus* biofilm formation as follows: (1) adhesion to an abiotic surface, (2) the development of a monolayer, (3) microcolony formation, (4) biofilm maturation, and (5) dispersal. Initial *S. aureus* adhesion is reversible via van der Waals forces, gravitational forces, surface electrostatic charge, hydrophobic interactions, Lewis acid–base interactions, and hydrogen bonding. *S. aureus* surface adhesion occurs via recognition of adhesive matrix molecules (MSCRAMMs), including FnBPa, FnBPB, and ClfA, by microbial surface compounds and via cell surface proteins, e.g., Bap, SasC, SasG, and Atl. D-AAs may be able to inhibit initial adhesion, reduce cell adhesion efficiency, and block bacterial attachment and growth from the foci of the monolayer. Robust aggregations composed of eDNA, amyloid fibers, polysaccharide intercellular adhesin/poly-ß (1-6)-N-acetylglucosamine (PIA/PNAG), polysoluble modulins (PSMs), and other proteins are formed. Some D-AAs may prevent this cell‒cell adhesion, leading to structural complications in the mature biofilm. During maturation, activated Agr-mediated quorum sensing (QS) initiates biofilm matrix modulation, and the EPS matrix is fully developed. QS can be activated either through PSM production or protease activation. PSMs maintain biofilm structure, and persister cells develop. D-AAs may interfere at this stage by incorporating into the peptidoglycan bond, inhibiting protein binding to the cell wall and disrupting cell‒cell and cell-surface interactions, thereby disassembling biofilm structure in areas of high concentration and/or preventing protein synthesis that is necessary for biofilm maintenance. This may enhance the effects of antibiotics. During dispersal, Agr-mediated QS initiates the dispersal of a segment of biofilm cells. This action is dependent on cell density signal molecules, namely, autoinducers. Autoinducing peptide (AIP) binds and activates histidine kinase (AgrC), which in turn phosphorylates AgrA. AgrA activates the transcription and production of a regulatory RNA molecule that impacts cell‒cell adhesion. This involves the release of PSMs, proteases and nucleases that aid dispersion. When the segment of cells becomes detached from the biofilm, they become planktonic and repeat the cycle, thereby infecting distant sites. During this phase, D-AAs may be capable of decreasing the metabolic activity and growth of planktonic cells

*Staphylococcus aureus* (*S. aureus*) is a nosocomial pathogen known to cause a variety of human disease conditions. It can act as a commensal, colonizing the skin and mucous membranes, and as a pathogen due to its invasive capacity; *S. aureus* is the most common microorganism isolated with PJIs.^179^ Furthermore, *S. aureus* biofilms have been implicated as a major cause (50%–60% of cases) of bone fracture non-union^180,181^ and play a critical role in the development of chronic osteomyelitis and a sequelae of infectious complications.^182–184^ Remarkably, the biofilm itself has been shown to have the ability to directly resorb bone.^185^ A major constituent in both *S. aureus* and *S. epidermidis* biofilms is polysaccharide intercellular adhesin (PIA), which plays an important role in structural integrity.^186^ *S. aureus* produces biofilms via both PIA-dependent and PIA-independent methods.^187^ Other major polysaccharides include capsular polysaccharide and cell wall teichoic acid. Furthermore, eDNA and global regulators, including *sarA, agr*, and *sigB*, play a role in the regulation of biofilm formation.^187,188^ Hochbaum and colleagues^169^ demonstrated that D-Tyr, D-Pro, and D-Phe, when administered at a concentration of 500 μmol·L^−1^, efficiently inhibited *S. aureus* biofilm formation. An equimolar mixture was shown to be even more potent and effectively inhibited biofilm formation at a concentration of 100 μmol·L^−1^ after 24 h of incubation. This study also reported that the production and localization of exopolysaccharide were not significantly affected; however, the D-AA mixture was able to disassemble preformed *S. aureus* biofilm, but only at the higher concentration of 10 mmol·L^−1^. Further investigation of protein surface localization in *S. aureus* in either L- or D-amino acid-treated cultures showed that the cells in biofilm aggregates formed in the L-AA cultures were clearly decorated with protein. However, there was a lack of this protein surrounding the cells in the D-AA cultures, suggesting a functional relationship between the effect of D-AAs and a protein component of the matrix. Several surface proteins, including Bap,^189^ SasG,^190^ FnBPA and FnBPB,^191^ and SasC,^192^ play an important role in *S. aureus* biofilm aggregation, and the authors speculated that D-AAs may prevent the localization of cell‒cell adhesion proteins, thereby inhibiting biofilm formation and development. However, in contrast, Sarkar and Pires^193^ reported no inhibitory effect on biofilm formation by D-Tyr, or a D-Tyr/D-Pro/D-Phe mix, when investigated at concentrations of 1 and 5 mmol·L^−1^ following a 24 and 48 h incubation period. Nevertheless, Sanchez Jr et al.^194^ demonstrated that 5 mmol·L^−1^ concentrations of D-Met, D-Phe, D-Pro, and D-Trp were each highly effective at preventing and disassembling *S. aureus* and MRSA biofilms, and this effect was augmented when the D-AAs were combined in vitro. The D-AAs displayed no significant effect on the growth of the bacteria. The team then demonstrated that polyurethane scaffolds incorporated with the D-1:1:1 mixture at 5 or 10 wt% significantly reduced *S. aureus* contamination in a rat segmental femoral defect model in vivo. Harmata et al.^195^ investigated the role of a 1:1:1 mixture of D-Pro/D-Met/D-Phe in inhibiting methicillin-sensitive *S. aureus* and MRSA biofilm formation and dispersal in vitro and in an ovine model in vivo. The results demonstrated that the D-AAs inhibited biofilm formation in both cases at concentrations of 13.5 mmol·L^−1^ per liter or greater after 24 h and that concentrations above 27 mmol·L^−1^ per liter significantly inhibited bone marrow stromal cell proliferation and osteoblast and osteoclast differentiation in vitro. Their findings also showed that the local delivery of 200 mmol·L^−1^ per liter D-Pro/D-Met/D-Phe released from low-viscosity calcium phosphate-based scaffolds did not inhibit new bone formation when injected into femoral condyle defects in vivo and 4 months following surgery. Li et al.^196^ investigated the effect of the D-AAs D-Phe, D-Pro, and D-Trp when combined with vancomycin in a rat model of *S. aureus* PJI. The authors demonstrated that lower concentrations of D-AAs (0.5 or 1 mmol·L^−1^ and not 10 mmol·L^−1^), when injected into the articular cavity of the knee weekly over a 6-week study period, exerted the least negative impact on the local distal femoral area and width. These findings demonstrated that a D-AA-vancomycin combination therapy resulted in high infection clearance, more so than vancomycin alone. Remarkably, the authors also reported that the combination was more effective at redressing the abnormal bone formation associated with the infection. Significantly increased levels of bone mineral density, bone volume, and trabecular thickness were reported, together with reduced levels of osteoclastic activity and increased tissue expression of osterix compared with vancomycin alone-treated animals and controls. Finally, the group of animals treated with a D-AA-vancomycin combination sustained normal weight gain and exhibited reduced levels of α2 M, IL-1β, IL-6, IL-10, TNF-α and PGE2 within serum when compared with the vancomycin-alone and control groups. Sanchez et al.^197^ investigated the effect of D-Met, D-Phe, and D-Trp on the disassembly of late-stage biofilms derived from clinical isolates of *S. aureus*. Their findings showed that concentrations of ≥5 mmol·L^−1^ effectively and significantly disassembled preformed biofilms and that when combined as an equimolar mixture, the anti-biofilm activity was further augmented. When combined with rifampin, the synergistic effect further amplified the inhibitory activity 2- to 4-fold to near bactericidal levels. This study also reported no effect of D-AAs on planktonic *S. aureus*. In contrast, Yang et al.^198^ demonstrated that D-Asp, when given at concentrations >10 mmol·L^−1^, significantly inhibited the growth of planktonic MRSA N315 in a time-dependent manner (6–112 h) and at subinhibitory levels, significantly decreasing its metabolic activity without influencing growth. The authors speculated that the decreased metabolic activity may have resulted in the decreased levels of protein and DNA measured in the matrix of biofilms formed in the presence of D-Asp. This study also showed that both D- and L-Asp at a concentration of 0.625 mmol·L^−1^ inhibited MRSA N315 biofilm formation on tissue culture plates by 42% after 24 h, and at a concentration of 0.5 mmol·L^−1^, they inhibited biofilms by 74% and 64% after 48 and 72 h, respectively. The D- and L-isoforms were found to be equally effective. At concentrations of 1 and 5 mmol·L^−1^, D-Asp was also able to disassemble 48 h aged preformed N315 biofilms. Finally, Wickramasinghe and colleagues^199^ recently investigated a 200 mmol·L^−1^ mixture of D-Tyr:D-Trp:D-Phe in a 1:22:57 molar ratio within a thermoresponsive hydrogel nanocomposite system. Developed as a novel PJI treatment approach, the study showed effective disruption and total eradication of *S. aureus* biofilms formed on titanium-, tantalum-, and cobalt chromium-based metal surfaces in vitro. Together, these preliminary studies highlight the important role of D-AAs and their significant promise as a novel approach in the prevention and treatment of *S. aureus*-infected PJIs (Figs. 4 and 5).

##### Staphylococcus epidermidis

*Staphylococcus epidermidis* is the most frequently isolated member of the group of coagulase-negative staphylococci in PJIs.^65^ Coagulase-negative staphylococci are associated with nosocomial acquired infections, and although less virulent than *S. aureus*, these bacteria are an important reservoir of antimicrobial resistance genes and resistance-associated mobile genetic elements that can be transferred between staphylococcal species.^200^ Generally, the success of *S. epidermidis* as a pathogen has been attributed to its ability to prolong adherence to surfaces and its ability to quickly form biofilms.^201^ The process of *S. epidermidis* biofilm formation is similar to that of *S. aureus*. Initial attachment is mediated by eDNA and proteins, including assembly activating protein (Aap), the autolysin AtlE, and the autotransporter protein Aae; the attachment occurs via microbial surface component recognition of adhesive matrix molecules.^202^ The protein Aap has a peptidoglycan binding motif and undergoes polymerization to form fibers;^203^ thus, the polymerization ability of Aap directly contributes to biofilm assembly/disassembly. Similar to *S. aureus*, biofilm dispersal occurs with the assistance of *agr* and is not yet fully understood.^204^ Varying levels of sensitivity to D-AAs have been demonstrated in a wide range of pathogenic and commensal *S. epidermidis* strains. Biofilm formation of 31 clinical isolates obtained from either healthy skin, conjunctiva, or ocular pathogenic infection showed varying levels of inhibition following the administration of D-Leu (17–50 mmol·L^−1^), D-Tyr (6–50 μmol·L^−1^), D-Pro (3–10 μmol·L^−1^), D-Phe (3–20 μmol·L^−1^), D-Met (15–50 mmol·L^−1^), or D-Ala (15–100 mmol·L^−1^).^205^ D-Met inhibited most of the 31 strains investigated (26/31), followed by D-Phe (21/31), after 24 h of incubation. No correlations between strain source and D-AA sensitivity were found in this study, and no inhibitory role was measured in the control L-isomer groups. When D-Pro/D-Met/D-Phe were mixed, a synergistic effect in biofilm inhibition was observed in the sensitive strains and the combination was also able to disassemble mature biofilm in some strains (10/31). The mechanism(s) involved remain unclear; however, the authors speculated that the D-AAs may interact with Aap or Embp in a similar manner to that observed in *B. subtilis* with the TasA protein.^117^ Aap consists of an LPXTG motif, which anchors it to the peptidoglycan in the cell wall, thereby providing the potential for D-AA-induced disruption and disassembly. In contrast, Sarkar and Pires^193^ found no inhibitory effect on *S. epidermidis* when D-Tyr or a D-Tyr/D-Pro/D-Phe mix was administered at concentrations of 1 and 5 mmol·L^−1^ following a 24 and 48 h incubation period. Future studies are warranted to elucidate the role of Aap and the interaction of D-AAs with other components found within the *S. epidermidis* biofilm structure.

##### Bacillus subtilis

Orthopedic implant-related *Bacillus spp*. infections are rarely reported, except after open fractures.^206,207^ However, concerns have been raised that diagnosis may go undetected when traditional culture analysis methods are used and that some patients with *Bacillus spp*. PJI subsequently require revision surgery.^208^ For example, *Bacillus cereus* was confirmed in a very late PJI, 13 years after THA, and in the absence of recent trauma or intervention.^209^ *Bacillus cereus* has also been confirmed as the pathogen in septic arthritis.^210,211^ *Bacillus subtilis* is a gram-positive, aerobic, rod-shaped, spore-forming bacterium, a ubiquitous organism, and a robust model organism to study the traits and molecular mechanisms of biofilm formation.^117^ Several studies have investigated the activity of D-AAs on *B. subtilis* and thus these studies are the focus of this section. When in biofilm, *B. subtilis* cells are enclosed and held in place by an extracellular matrix consisting of cell-anchored amyloid fibers composed of the amyloidogenic protein TasA^212^ and hydrophobic BslA proteins.^213–215^ TasA is encoded by the *tasA* operon epsA-O. Deletion of the *pgcA* and *gtaB* genes leads to impaired biofilm formation.^213,216,217^ The TasA amyloid fibers provide structural integrity to the biofilms, and as biofilms senesce, they fall apart as the fibers are released from the cell.^215^ Loss of BslA results in a reduction in surface repellency and an altered biofilm surface microstructure.^214^ Additional proteins are necessary for the polymerization of these fibers, and TapA has been shown to contribute to the assembly of TasA and the transition into the fiber state, and the signal peptidase SipW processes both proteins into their mature forms.^218^ During the late stages of a biofilm life cycle, cells begin to produce a mixture of D-AAs, including D-Tyr, D-Met, D-Leu, and D-Trp, at a concentration of 3 μmol·L^−1^.^215^ As these D-AAs are incorporated into the peptidoglycan, TasA amyloid fibers are released from the cells, leading to biofilm disassembly. Romero et al.^215^ showed that TapA is found in discrete foci in the cell envelope, and these foci disappear when cells are treated with a mixture of D-AAs, suggesting that TapA may be a key target for therapeutic D-AA delivery. Similarly, Kolodkin-Gal et al.^118^ found that D-Tyr, D-Leu, D-Trp, and D-Met actively inhibited *B. subtilis* biofilm formation, both in liquid medium and on a solid surface. Interestingly, the individual D-AA concentrations required to effectively inhibit biofilm formation varied with the D-AAs as follows: D-Tyr (3 μmol·L^−1^), D-Met (2 mmol·L^−1^), D-Trp (5 mmol·L^−1^), and D-Leu (8.5 mmol·L^−1^). The mixture of all four D-AAs was significantly more potent, suggesting synergistic activity. D-Tyr and the mixture D-AAs caused pellicle breakdown and disassembled the preformed biofilm. The specific mechanism(s) remains elusive. The D-AAs did not inhibit the growth or expression of the matrix operons, but substitution of D-Tyr with D-Ala prevented these effects, suggesting that they may act through their incorporation into the peptidoglycan cell wall. However, it has been subsequently reported that the *B. subtilis* strain investigated contained a mutation in the *dtd* gene, the D-tyrosyl-tRNA deacylase that makes proteins receptive to D-AA incorporation.^219^ Complementation with the wild-type Dtd enzyme made *B. subtilis* resistant to the anti-biofilm activity of D-AAs.^108^ Yu et al.^171^ investigated a range of D-Tyr concentrations on *B. subtilis* and showed that it inhibited biofilm formation at the low, sublethal concentration of 5 nmol·L^−1^ and at the higher concentration of 200 μmol·L^−1^, with no effect on growth; the amount of biomass slightly increased at intermediate concentrations ranging between 1 and 10 μmol·L^−1^. Compared to *P. aeruginosa*, the effect of D-Tyr delivered a more potent inhibitory effect in *B. subtilis*, and biofilm formation was reduced 42% with 5 nmol·L^−1^ D-Tyr and when compared to the control and with changes in EPS composition. The specific effects on extracellular protein and polysaccharides varied depending on the D-Tyr concentration. Preformed *B. subtilis* biofilms detached more easily in the presence of D-Tyr or a mixture of D-Tyr, D-Met, D-Trp, and D-Leu. Similarly, Kolodkin-Gal et al.^118^ demonstrated that a mixture of D-Tyr, D-Leu, D-Trp and D-Met inhibited *B. subtilis* biofilm formation at 10 nmol·L^−1^. Leiman et al.^219^ demonstrated inhibition of bacterial growth by D-Tyr at 6 μmol·L^−1^ and speculated potential misincorporation into protein during protein synthesis, resulting in proteotoxicity as a primary mode of action and thereby inhibiting biofilm formation in vitro. D-Tyr inhibited the expression of key biofilm matrix genes, *epsA* and *tapA*; interestingly, supplementation with L-amino acids specifically reversed the inhibitory effect of their cognate D-AAs. This study also demonstrated that both independently and as a mixture, D-Tyr, D-Leu, D-Met, and D-Trp inhibited growth in a similar way. A study by Bucher et al.^117^ investigated the effect of 0.5 and 10 mmol·L^−1^ D-Leu on *B. subtilis* and found no inhibitory effect on cell growth but D-Leu did impair biofilm formation, which was specifically due to cell wall interferences, including inhibition of peptidoglycan transpeptidation and transglycosylation or of wall teichoic biosynthesis. Further analyses showed that D-Leu altered the anchoring of the matrix amyloid TasA without affecting TasA transcription from its upstream promotor or its protein levels. Finally, Hochbaum et al.^169^ demonstrated that a mixture of D-Tyr, D-Pro, and D-Phe was more effective at inhibiting *B. subtilis* biofilm formation than the mixture of D-Trp, D-Met, D-Leu and D-Tyr previously reported to be efficacious by Kolodkin-Gal.^118^ In contrast, Sarkar and Pires^193^ found no inhibitory effect on *B. subtilis* when a D-Tyr or a D-Tyr/D-Pro/D-Phe mix was administered at concentrations of 1 and 5 mmol·L^−1^ and following a 24 and 48 h incubation period.

##### Streptococcus mutans

Although uncommon, the risk of PJI from hematogenous bacterial seeding is increased in patients undergoing dental procedures. Dental treatments able to facilitate the development of bacteremia have been identified; acute *S. mutans* or *S. salivarius* infections have been reported in THA and TKA,^220–222^ and *S. viridans* has been reported in PJI.^223^ *Streptococcus mutans* is a major cariogenic bacterium that plays a key role in the development of dental plaque.^224^ Dental plaque is a typical biofilm and is a matrix consisting of polysaccharides, proteins, and microbial cells. *Streptococcus mutans* utilizes extracellular sucrose to produce the exopolysaccharide glucan, which promotes the adhesion of microorganisms onto the tooth surface.^225^ *S. mutans* attaches through either sucrose-dependent or sucrose-independent mechanisms. In sucrose-dependent attachment, *S. mutans* utilizes three glucosyltransferases, GtfB, GtfC, and GtfD, to initiate biofilm formation with sucrose as the substrate. Following this step, glucan-binding proteins combine with glucosyltransferases and adhesive glucans to create a scaffold for the organism to attach to and produce biofilms.^226,227^ Sucrose-independent formation occurs through the use of antigen I/II, which serve as adhesins that bind to the desired surface.^226,228^ A limited number of studies have investigated the effect of D-AAs on *S. mutans*. Tong et al.^229^ investigated the effect of three amino acids either alone or in combination with the AMP nisin on planktonic *S. mutans* and *S. mutans* biofilms and showed that D-Cys, D- or L-Asp, and D- or L-Glu significantly improved the antibacterial activity of nisin against *S. mutans*. Furthermore, the mixture of D-Cys, D-Asp, and D-Glu and the mixture of L-Cys, L-Asp, and L-Glu at a concentration of 40 mmol·L^−1^ prevented *S. mutans* growth. The study also showed that D- or L-Cys, Asp, and Glu at a concentration of 40 mmol·L^−1^ and without nisin inhibited biofilm formation and were more potent when combined than when assessed individually. This study further showed that D-Cys, D-Asp, and D-Glu at a concentration of 10 mmol·L^−1^ did not improve the antibacterial activity of nisin, and a significant improvement was measured at concentrations of 40 mmol·L^−1^ only. However, D-Val, D-Phe, D-Leu, D-Ile, D-The, D-Pro, D-Tyr, and D-Ser showed no inhibitory effect when compared with the control at any of the concentrations investigated. Interestingly, the combination of L-Cys, L-Asp, and L-Glu exerted stronger antibiofilm activity than the D-AA combination. Nevertheless, further work to investigate the interaction of D-AAs with glucan-binding proteins, glucosyltransferases, and antigen I/II may aid in the development of novel technologies that not only reduce dental plaque and caries but may also play a role in regulating biofilms in PJI.

##### Enterococcus faecalis

Enterococci are reported as the causative pathogen of PJIs in 2.3%–11% of cases,^230,231^ commonly affecting elderly individuals, and enterococci infections are considered difficult to treat.^232,233^ When attached to a surface, enterococci form biofilms and have a high degree of antimicrobial resistance.^234^ The gram-positive, diplococcus, facultative anaerobe *Enterococcus faecalis* is the most common species in PJI and accounts for 82%–85% of enterococcal infections, with 50%–64% presenting as polymicrobial infections.^235,236^ Furthermore, enterococci-infected implants have been associated with a higher failure rate than both staphylococcal and streptococcal PJIs.^233,237,238^ Several virulence factors are related to *E. faecalis* biofilm formation. Enterococcal surface protein (esp) has been demonstrated to play a primary role in cell adhesion and the colonization of abiotic surfaces.^239,240^ Furthermore, gelatinase (gelE), an extracellular metalloprotease that hydrolyzes gelatin, collagen, and hemoglobin, is also reported to be pivotal in *E. faecalis* adhesion and biofilm formation.^241^ Furthermore, gelE has recently been shown to be key in degrading the inducible antimicrobial peptide cecropin, which is known to perform a critical role in host defense.^242^ Chuang-Smith et al.^243^ reported that aggregating substance (agg) promoted biofilm formation in an ex vivo model of cardiac valve colonization, and Afonina et al.^244^ recently demonstrated that agg together with biofilm-associated pili (Ebp), when at higher cell densities, worked synergistically to promote maximal biofilm strength. However, the association of virulence factors involved in *E. faecalis* largely remains unknown. From the initial 37 *E. faecalis* clinical strains, Zilm et al.^245^ chose the 10 most potent biofilm producers and investigated the effect of a D-AA mixture of D-Leu, D-Met, D-Tyr, and D-Trp (concentrations of 0.25, 2.5, or 25 mmol·L^−1^) and their cognate L-isomers to reduce preformed biofilm over 24, 72, and 144 h in vitro. Remarkably, the study showed that the D-AAs significantly reduced biofilm formation in all strains in a dose-dependent manner and to a greater degree than the L-AA mixture. When the D-AAs at concentrations of 0.25 and 2.5 mmol·L^−1^ were tested on a planktonic culture, no significant reduction was measured. However, the addition of a 25 mmol·L^−1^ dose significantly reduced *E. faecalis* growth. Rosen et al.^246^ demonstrated that a 2 mmol·L^−1^ concentration of D-Leu was effective in disassembling *E. faecalis* preformed biofilms on dentin slabs without disrupting planktonic growth ~10-fold when compared with the control group. Few studies have investigated the promising effect of D-AAs on *E. faecalis* biofilm disassembly, and further work to investigate the effect on esp, gelE, agg, and Ebf may further elucidate the mechanistic role of D-AAs on *E. faecalis*, as well as improve our knowledge of the adhesion and aggregation proteins involved in *E. faecalis* biofilm formation.

#### Gram-negative organisms in PJI and the effect of D-AAs

##### Pseudomonas aeruginosa

*Pseudomonas aeruginosa* is a gram-negative rod-shaped microorganism that is typically found on the skin and in aquatic environments. *P. aeruginosa*-infected PJIs are considered to be one of the most difficult to treat due to the growing rate of multidrug-resistant strains and their ability to develop virulence and persistence mechanisms, such as biofilm formation and the production of small colony variants.^247,248^ Furthermore, *P. aeruginosa* has the propensity to attach to bone and fibrocartilaginous articular structures and is associated with osteomyelitis and septic arthritis.^249,250^ It is the cause of 5%–20% of gram-negative infections, with an incidence of 14% in patients with an open fracture.^251,252^ The reported treatment success rates with early, late-chronic or hematogenous *P. aeruginosa* PJIs following the use of debridement and implant retention or 2-stage exchange surgery range between 66% and 85%.^59,253–255^ Due to their wide occurrence, *P. aeruginosa* biofilms have been extensively studied. *P. aeruginosa* uses flagella to swim to, locate, and adhere to the implant surface^256,257^ and produces multiple EPSs, including Pel, Psl, and alginate. These polysaccharides differ in chemical structure and in their biosynthetic mechanisms.^258^ Pel and Psl help maintain cell-to-cell interactions, with alginate performing a similar role in strains isolated from mucoid variants. Extracellular DNA, extracellular type IV pili, and flagella are also known to be involved in initiating biofilm formation.^259,260^ Yu et al.^171^ investigated a range of D-Tyr concentrations, and when administered to *P. aeruginosa*, the results showed that it was able to inhibit biofilm formation at both low, sublethal concentrations of 5 nmol·L^−1^ and at higher concentrations of 200 μmol·L^−1^. In contrast, a slightly increased biomass was measured at intermediate concentrations ranging between 1 and 10 μmol·L^−1^. The study found that D-Tyr did not promote the detachment of preformed *P. aeruginosa* biofilms from a glass surface. However, in contrast, at higher D-Tyr concentrations, Kao et al.^261^ investigated the effect of D-Trp (10 mmol·L^−1^) and D-Tyr (1 and 10 mmol·L^−1^) on *P. aeruginosa* PAO1 biofilm formation and determined that biofilm formation was not inhibited by these D-AAs. Furthermore, D-Ala, D-Leu, and D-Met were also investigated at 10 mmol·L^−1^ concentrations, and no beneficial effects were reported. However, Rumbo et al.^174^ and using the same bacterial strain and at similar concentrations of 4 and 6 mmol·L^−1^, showed inhibition of biofilm formation. The reason for these differences remains unclear. A 4 mmol·L^−1^ concentration of D-Cys, D-Tyr, and D-Trp produced the highest inhibitory effects, causing a 30%, 16%, and 10% reduction in biofilm, respectively. Interestingly, D-Ala, D-Gln, and D-Arg stimulated *P. aeruginosa* growth and induced a 10%–40% increase in biofilm formation, while growth was not affected by D-Cys or D-Tyr; these results show the differences in how bacteria respond to the varying D-AAs, similar to work reported by He et al.^262^. In terms of anti-virulent activity in vitro, D-Trp, D-Cys, and D-Arg resulted in a decrease in the virulence of *P. aeruginosa* and increased the survival of A549 alveolar cells by 56%–45%. Despite this in vitro activity, no significant effect against *P. aeruginosa* infection was measured when investigated in an in vivo murine model. Sanchez et al.^197^ investigated D-Met, D-Phe, and D-Trp at concentrations of ≥5 mmol·L^−1^ and reported each effectively and significantly disassembled preformed biofilms of *P. aeruginosa* clinical isolates. This effect was further enhanced when an equimolar mixture (D-Met/D-Phe/D-Trp) was applied. Interestingly, the addition of the D-AAs also enhanced the activity of colistin and ciprofloxacin against biofilms of *P. aeruginosa*, reducing levels of viable bacteria >2 logs and 1 log, respectively, when compared with when the antibiotics were given alone. However, the activity of these antimicrobials was not enhanced when combined with the D-AAs and applied to planktonic cells. Finally, Brandenburg et al.^263^ investigated the effect of D-Trp, D-Tyr, D-Met, and D-Leu at concentrations ranging between 0.5 and 10 mmol·L^−1^ on *P. aeruginosa* biofilm formation. The results showed that at a concentration of 10 mmol·L^−1^, D-Trp and D-Tyr inhibited biofilm formation, but D-Met and D-Leu had no effect. D-Trp was most effective at the higher concentration of 10 mmol·L^−1^ and reduced biofilm formation by 71% at 24 h and 78% at 48 h following supplementation. Interestingly, and in contrast to the results reported by Kolodkin-Gal et al.,^118^ when both L- and D-Trp were mixed in an equimolar ratio, *P. aeruginosa* biofilm was inhibited by 93% at 24 h and 90% at 48 h. The L- and D- mixes inhibited bacterial growth and disassembled biofilms more robustly after 72 h of incubation, with limited disassembly measured when investigated at the lower concentration of 1 mmol·L^−1^. Finally, D-Trp significantly increased swimming and twitch motility, which also suggests implications in biofilm formation, as there is an inverse relationship between bacterial motility and biofilm formation,^264^ and flagellar arrest is required for biofilm formation.^265^

##### Acinetobacter baumannii

*Acinetobacter baumannii* is an aerobe gram-negative rod-shaped species that typically colonizes the skin surface and is commonly found in nosocomial hospital environments.^266^ Infections caused by *A. baumannii* are considered a serious health care threat because they are associated with the chronic colonization of human tissues and persistence on implanted medical devices.^174,267,268^ Infections caused by *A. baumannii* represent ~2% of all healthcare-associated infections in the U.S. and Europe,^269^ and globally, ~45% of all *A. baumannii* isolates are multidrug resistant, further complicating clinical outcomes.^270,271^ The rate of orthopedic implant-associated *A. baumannii* infections range from 0.6%–28.7%,^272–274^ with a mortality rate of 30.7%,^274,275^ and these infections are an increasingly common cause of osteomyelitis and delayed healing in soldiers with orthopedic battlefield wounds.^276–278^ Furthermore, a recent study by Choe et al.^268^ showed that *A. baumannii* inhibited implant osseointegration when investigated in a murine model of infection. An estimated >75% of all isolates are capable of forming biofilms, and the important role of biofilm-associated protein (Bap), a surface protein that facilitates adhesion and confers structural integrity to the biofilm, has recently been described.^279^ However, the mechanisms of biofilm formation remain mostly unknown. Other important factors include CsuE, OmpA, and class A extended beta-lactamase blaPER-1.^267^ CsuE is predominant in pili, thereby contributing to adherence; OmpA is a porin involved in species attachment and drug resistance; and Beta-lactamase blaPER-1 is also involved in cell adhesion. Although the specific mechanism(s) remain to be discovered, Rumbo and colleagues^174^ demonstrated that 1 mmol·L^−1^ D-AAs D-Cys, D-Trp, and D-His were most effective against *A. baumannii* growth (mainly D-Trp and D-Cys), biofilm formation and attachment to eukaryotic cells. Equimolar concentrations of 4 mmol·L^−1^ D-Cys/D-Trp, D-His/D-Thr/D-Trp/D-Ser/D-Arg/D-Glm and D-His/D-Thr/D-Trp/D-Ser/D-Cys yielded levels of inhibition in biofilm formation of 95%, 59%, and 58%, respectively. However, none of the D-AAs were able to protect against infection in vitro or in a murine model in vivo, and D-AAs may not be suitable anti-virulence agents. A recent study by Jariyarattanarach et al.^280^ reported the creation of a novel hybrid D-AA of modified aurein and cathelicidin, where the structures were substituted with hydrophobic and positively charged Trp and Arg. The hybrid D-AA exhibited potent antibacterial activity against *A. baumannii* and killing *via* membrane disruption and leakage of intracellular contents with a low tendency to induce bacterial resistance. Remarkably, the hybrid D-AA demonstrated potent activity against both multidrug- and extensively drug-resistant clinical isolates of *A. baumannii*.

## Role of D-AAs in eukaryotic tissues and their role in bone tissue turnover

Although not the main focus of this review, D-AAs also play a significant role in eukaryotic organisms, and their role in bone tissue homeostasis remains largely unexplored (Table 2). Host-synthesized D-Ser, D-Asp, D-Ala, and D-Cys have been identified in mammalian tissues, while the gut microbiota is composed of a great diversity of commensal bacterial species that also release and regulate abundant and diverse D-AAs.^281,282^ Notably, D-AAs have recently been associated with mucosal homeostasis.^281^ D-Ser localizes in astrocytes and neurons, and these cells are able to both synthesize and degrade D-Ser;^283,284^ hence, D-Ser is present in various regions of the brain.^283,285^ N-methyl-D-aspartate (NMDA) receptors are associated with learning and memory, and D-Ser (also D-Asp and D-Ala) is able to bind to NMDA receptors.^286^ A recent study by Beltran-Castillo et al.^287^ demonstrated that astrocytes in the mouse caudal medullary brainstem synthesized, stored, and released D-Ser in response to elevated CO_2_ levels. Remarkably, through D-Ser binding to NMDA receptors, the breathing response to CO_2_ levels was directly regulated. While increased levels of D-Ser potentiate glutamate transmission, thereby increasing synaptogenesis and synaptic plasticity, D-Ser also appeared to potentiate NMDA receptor-dependent excitotoxicity, promoting neurodegeneration and cognitive impairment.^288^ These mechanisms could also be involved in neurodegenerative diseases,^289^ and indeed, increased levels of D-Ser have been measured in the brain tissue,^290^ blood, and cerebrospinal fluid^291,292^ isolated from patients with Alzheimer’s disease. As such, D-Ser is considered an important contributor in regulating the NMDA receptor-mediated neurotoxic changes that lead to Alzheimer’s disease and may also play a major role in the development of schizophrenia and epilepsy.^293,294^ Much remains to be discovered about the role of D-Ser in bone tissue. D-Ser, *via* NMDA receptor mediation, is secreted by osteoblasts and does not appear to affect osteoblastogenesis, but a paracrine effect of osteoblast-derived D-Ser on neighboring osteoclasts has been proposed. Takarada et al.^295^ identified the expression of serine racemase mRNA in osteoblasts localized on the cancellous bone surface in neonatal rat tibial sections. This study reported that sustained exposure to cultured calvarial osteoblasts in vitro did not affect alkaline phosphate levels or Ca^2+^ accumulation but significantly inhibited osteoblast maturation in a dose-dependent manner without affecting the survival of osteoclasts. The authors also reported that D-Ser negatively regulated osteoclastogenesis from bone marrow-derived precursors, which may play a pivotal role in inhibiting the bone resorption process. However, the mechanisms for this remain elusive. Rivera-Villasenor et al.^296^ further theorized that the expression of NMDA receptors on early osteoblasts decreases with increasing age, which may subsequently inhibit osteoblast maturation. The authors speculated that this would result in a secondary decrease in D-Ser release by mature osteoblasts, thereby promoting osteoclast maturation, bone resorption, and potentially progressing age-associated osteoporosis.

**Table 2:** A comparative table displaying the differing roles of D-AAs in prokaryotes versus eukaryotes, with a focus on their role in bone tissue

D-Amino Acid	Role in Prokaryotes	Role in Eukaryotes and Bone
D-Alanine	• Peptidoglycan component^68,121^ • Modulation of peptidoglycan structure and synthesis^215^ • Anti-germination properties to bacterial spores^136^ • Provides cell wall resistance to protease activity^68,121^	• Hypothesized role in nociception in bone cancer^308^ • High levels associated with diseases of aging, primarily deposits in bone, skin, arteries, and lenses • Potential anticancer properties through the induction of cytotoxic H_2_O_2_ synthesis of D-AA oxidase^309^ • Association of high levels with gastric cancer^305,306^ • Association of high levels with kidney disease and impaired renal function^313,314^
D-Arginine	• Modulation of bacterial virulence^174^	• Eukaryotic cell protection from cell death in bacterial infections^174^
D-Aspartic Acid	• Protection from bactericidal agents (e.g., vancomycin) as a terminal residue of stem peptides^108,112,124–127^ • Inhibition of bacterial adhesion to abiotic surfaces^172^ • Inhibition of biofilm formation^198,229^ • Inhibition of planktonic MRSA growth^198^ • Inhibition of metabolic activity^198^ • Inhibition of bacterial growth^229^	• D-Asp-NMDA receptor expression associated with osteoblast and osteoclast activity in vitro^300^ • Regulation of collagen type I, osteocalcin, and alkaline phosphatase levels in osteoblasts^301^ Increased levels in Paget’s disease and osteoporosis^302^ • Hypothesized association with oxidative stress and aging disorders, with deposits mainly found in bone, skin, arteries, and lenses^299^ • Neurotransmission^297^
D-Cysteine	• Inhibition of bacterial adhesion to eukaryotic cells^174^ • Inhibition of bacterial growth^174,229^ • Inhibition of biofilm formation^174,229^ • Modulation of bacterial virulence^174^	• Eukaryotic cell protection from cell death in bacterial infections^174^
D-Glutamic Acid	• Peptidoglycan component^68,121^ • Inhibition of bacterial growth^229^ • Inhibition of biofilm formation^229^ • Provides cell wall resistance to protease activity^68,121^	• Association with cataracts and deposition within the lens^304^ • Association of low levels with hepatocellular carcinoma^307^
D-Histidine	• Anti-germination properties to bacterial spores^138^ • Trapping and sequestering of metals for bacterial growth^143^ • Inhibition of biofilm formation^174^ • Inhibition of bacterial adhesion to eukaryotic cells^174^ • Inhibition of bacterial growth^174^ • Modulation of bacterial virulence^143^	• Unknown
D-Leucine	• Inhibition of biofilm formation^108,118^ • Biofilm disassembly^245,246^ • Modulation of peptidoglycan structure and synthesis^215^ • Host immune evasion and suppression by activation of sweet taste receptors^147^ • Inhibition of bacterial adhesion to abiotic surfaces^172^ • Inhibition of bacterial growth^118^	• Unknown
D-Methionine	• Modulation of peptidoglycan structure and synthesis^215^ • Inhibition of bacterial adhesion to eukaryotic cells^174^ • Inhibition of biofilm formation^118,194,195^ • Biofilm disassembly^194,197,245^ • Inhibition of bacterial growth^118^	• Bone formation at low concentrations (≤50 mmol·L^−1^); inhibition of bone marrow stromal cell proliferation, and osteoblast and osteoclast differentiation at high concentrations (>12.5 mmol·L^−1^)^195,263,319^ • No inhibition of new bone formation in an in vivo ovine model^195^ • No adverse bone tissue response in a rat segmental defect model in vivo^194^
D-Phenylalanine	• Inhibition of biofilm formation^108,169,194,195^ • Biofilm disassembly^169,194,197^ • Host immune evasion and suppression by activation of sweet taste receptors^147^ • Modulates peptidoglycan synthesis and strength^113^	• Bone formation at low concentrations (≤ 50 mmol·L^−1^); inhibition of bone marrow stromal cell proliferation, and osteoblast and osteoclast differentiation at high concentrations (>12.5 mmol·L^−1^)^195,263,319^ • Increased bone density, volume, trabecular thickness, and reduced osteoclastic activity in vivo^196^ • Reduced *S. aureus*-induced abnormal bone remodeling^196^ • No inhibition of new bone formation in an in vivo ovine model^195^ • No adverse bone tissue response in a rat segmental defect model in vivo^194^
D-Proline	• Inhibition of biofilm formation^169,194,195^ • Biofilm disassembly^169,194^	• Bone formation at low concentrations (≤ 50 mmol·L^-1^); inhibition of bone marrow stromal cell proliferation, and osteoblast and osteoclast differentiation at high concentrations (>12.5 mmol·L^-1^)^195,263,319^ • Increased bone density, volume, trabecular thickness, and reduced osteoclastic activity in vivo^196^ • No inhibition of new bone formation in an in vivo ovine model^195^ • Reduced *S. aureus*-induced abnormal bone remodeling^196^ • No adverse bone tissue response in a rat segmental defect model in vivo^194^
D-Serine	• Protection from bactericidal agents (e.g., vancomycin) as a terminal residue of stem peptides^108,112,124–127^ • Upregulation of virulence gene expression and bacterial colonization^144^ • Cell growth^144^• Inhibition of bacterial adhesion to eukaryotic cells^145,174^	• Secreted by osteoblast. Modulates osteoblast maturation and osteoclastogenesis in bone^295,296^ • Neurotransmission. Regulation of N-methyl-D- aspartate receptor activity^286^ • Association with Alzheimer’s disease^290–292^ • Association with cataracts^304^ • Association of high levels with kidney disease and impaired renal function^313,314^
D-Tryptophan	• Host immune evasion^150^ • Inhibition of bacterial adhesion to abiotic surfaces^172^ • Inhibition of biofilm formation^118,174,194,263^ • Biofilm disassembly^194,197,245^ • Inhibition of bacterial growth^118,174^ • Modulation of bacterial virulence^174^ • Promotion of bacterial motility (swimming and twitching)^263^ • Inhibition of bacterial adhesion to eukaryotic cells^174^	• Increased bone density, volume, trabecular thickness, and reduced osteoclastic activity in vivo^196^ • Reduced *S. aureus*-induced abnormal bone remodeling^196^ • No adverse bone tissue response in a rat segmental defect model in vivo^194^ • Eukaryotic cell protection from cell death in bacterial infections^174^
D-Tyrosine	• Inhibition of biofilm formation^118,169,171^ • Biofilm disassembly^169,245^ • Inhibition of bacterial adhesion to abiotic surfaces^170–173^ • Increased surface detachment of biofilm^171^ • Inhibition of bacterial growth^118^ • Modulates peptidoglycan synthesis and strength^113^	• Unknown
D-Valine	• Inhibition of bacterial adhesion to eukaryotic cells^174^	• Unknown

Ball and Stick components are indicated as follows: red = oxygen, blue = nitrogen, yellow = sulfur, and white = hydrogen. Information was sourced from the National Library of Medicine, National Center for Biotechnology information https://pubchem.ncbi.nlm.nih.gov

D-Asp is also found in the central nervous system and appears to play a fundamental role in neurotransmission,^297^ as well as in endocrine organs, e.g., the pineal gland, pancreas, and adrenal gland.^286^ Notably, significantly reduced levels of D-Asp have been measured in the prefrontal cortexes of patients with schizophrenia.^298^ Furthermore, significantly higher levels of D-Asp have been measured in various tissues in elderly individuals (*e.g*., bone, skin tissue, lenses, and arterial walls).^299^ As nonenzymatic or spontaneous racemization is associated with aging and oxidative stress, increased D-Asp levels are considered to be related to old age and may be associated with several common aging disorders. In the context of bone, D-Asp-NMDA receptor expression is associated with both osteoblast and osteoclast activity in vitro.^300^ Ho et al.^301^ demonstrated that NMDA receptor mediation *via* its coagonist D-Asp regulated collagen type I, osteocalcin, and alkaline phosphatase levels in osteoblasts and may play an important role in transmitting mechanical load recognition in a rat model of disuse osteopenia. Significantly increased levels of D-Asp within urine samples of patients with Paget’s disease and osteoporosis have also been reported.^302^ D-Asp is also likely involved in aging of the skin and the development of arthrosclerosis, macular degeneration, and cataracts.^296,299,303^ To this end, several D-AAs have been identified in human lenses in the eye, including D-Asp, D-Ser, D-Glu/Gln, and D-Phe, where the amount of racemization of D-Ser and D-Asp was significantly increased in cataract lenses when compared with age-matched healthy lenses.^304^

In terms of cancer and tumor growth, D-AAs appear to display a varied response dependent on tumor etiology and condition. For example, significantly high levels of D-Ala have been measured in the gastric juice of gastric cancer patients,^305^ and as such, more recently, Zhang and colleagues^306^ developed a noninvasive luminescent DNA-silver nanocluster test to identify D-Ala in saliva for the early detection of gastric cancer. However, in contrast, Han et al.^307^ reported reduced levels of D-Glu and D-Glc in the serum of patients with hepatocellular carcinoma when compared to a healthy cohort. Huang et al.^308^ investigated the role of D-AA oxidase and its association with pain due to bone cancer. D-AA oxidase is almost exclusively expressed by astrocytes and distributed within the spinal cord. It catalyzes the oxidation of D-AAs to their corresponding α-keto acids, ammonia, and hydrogen peroxide (H_2_O_2_) and has been shown to be involved in chronic pain conditions.^309^ In this study, the authors demonstrated that in rats, inoculation of the tibia with mammary gland carcinoma cells produced mechanical allodynia, synchronous with the induction of D-AA oxidase expression and enzymatic activity. The intrathecal injection of a D-AA oxidase inhibitor blocked mechanical allodynia in a dose- and time-dependent manner, with a maximum inhibition of 40%–50%, indicating the first evidence that D-AAs may have a role in nociception. The authors suggested that this may be due to reduced spinal H_2_O_2_ levels, which inhibit astrocyte hypertrophy. Furthermore, due to the α-keto acid-, ammonia-, and highly damaging H_2_O_2_-induced oxystress nature of D-AA oxidase activity, this enzyme has been utilized in gene-directed enzyme prodrug therapies that target cancer. Oxidative damage to DNA, proteins and lipids on tumor cells promotes their apoptosis, and Rosini et al.^310^ showed that the cytotoxic effect of D-AA oxidase on mouse N2C mammary gland tumor cells, among various other cancer cell lines, was significantly increased at low local O_2_ concentrations representative of the tumor microenvironment following supplementation with D-Ala at the optimal concentration of 30 μmol·L^−1^. D-Ala contributed by beneficially modifying specific kinetic steps and thus improving enzyme activity and the cytotoxic effect, thus demonstrating that D-Ala may be a novel tool for cancer treatment that exploits the production of H_2_O_2_. More recent studies have used this D-Ala-mediated approach to inhibit angiogenesis and the proliferation of glioma cells^311^ or through the use of functionalized nanoparticle-induced cytotoxicity in ovarian adenocarcinoma cells.^312^

Interestingly, D-Ser, D-Asn, D-Ala, and D-Pro have been measured at significantly high levels in patients with kidney disease when compared to healthy people, and their levels correlated with kidney function.^313,314^ D-Ala has been identified in the human brain,^315^ and Tsai et al.^316,317^ reported that the addition of D-Ala or D-Ser to antipsychotics significantly improved the treatment of schizophrenia as early as 2 weeks following treatment. The authors suggested that the improvement occurred potentially via hypofunction of NMDA neurotransmission. Together, these studies briefly highlight the abundant role of D-AAs in human tissue, as well as the promising versatile roles of D-AAs as a novel future clinical therapy potentially able to treat a vast number of human conditions, as detailed in a recent thorough review by Shi et al.^318^. However, their role in bone regeneration, repair, and disease remains largely unexplored. Future studies in this area are of high importance and critical to further unveil the mechanistic insights required to support the development of future novel and improved orthopedic strategies to combat bone disease, including the challenges of PJIs.

## D-AA cytotoxicity

To be efficacious as a clinical therapy, D-AAs must confer limited cytotoxicity at concentrations that are effective in preventing bacterial adhesion to the orthopedic implant surface, inhibiting biofilm formation, and initiating the dispersal of established biofilm. A wide range of D-AA concentrations (5 nmol·L^−1^–40 mmol·L^−1^) has been assessed in this review, and clear discrepancies in efficacy highlight that further work is needed. Nevertheless, these differences are likely due to strain and species heterogeneity, as well as the differing types of D-AAs investigated. Notably, not all studies included parallel, host cell cytotoxicity analyses to allow for speculated potential clinical efficacy. The host cell response was investigated by Harmata et al.,^195^ who showed that a 13.5 mmol·L^−1^ equimolar mixture of D-Pro/D-Met/D-Phe was effective at inhibiting *S. aureus* biofilm formation and disassembly. Remarkably, osteoblast and osteoclast differentiation were only significantly inhibited at concentrations above 27 mmol·L^−1^ per liter, and bone marrow stromal cell proliferation was inhibited at a concentration of 54 mmol·L^−1^ in vitro. Sanchez et al.^197^ demonstrated that D-Phe, D-Pro, D-Met, and D-Trp prevented biofilm formation and activated biofilm dispersal of *S. aureus* at concentrations ≥5 mmol·L^−1^. Similarly, this study also showed that osteoblasts and fibroblasts treated with this D-AA mix for 24 h maintained >70% viability at concentrations ≤50 mmol·L^−1^, while D-Trp exhibited cytotoxic effects (i.e., <70% viability) at concentrations >12.5 mmol·L^−1^. However, Rawson et al.^319^ reported that 250–500 μmol·L^−1^ of a D-Pro/D-Tyr/D-Phe mixture reduced bone marrow-derived mesenchymal stem (BMSC) cell viability and alkaline phosphatase expression. Furthermore, Brandenberg et al.^263^ reported no toxicity of L- and D-Trp equimolar mixtures to HaCaT cells at concentrations between 1 and 10 mmol·L^−1^, concentrations where *P. aeruginosa* biofilm was effectively inhibited. However, the cytotoxicity of D-Phe, D-Met, and D-Trp to Chinese hamster ovary and HeLa cells at concentrations ≥10 mmol·L^−1^ has been reported.^320^

When investigated in vivo, no signs of systemic illness in rats post intra-articular injection of 10 mmol·L^−1^ of equimolar 1:1:1 D-Phe:D-Pro:D-Trp after a 6-week study period was reported by Li et al.^196^. Indeed, this study reported that the therapeutic effect of a D-AA-vancomycin mix significantly reduced *S. aureus*-induced abnormal bone remodeling when compared to rats given vancomycin alone. However, dose-dependent impairment of bone architecture was also reported in this study. Harmata et al.^195^ reported no adverse effects and that the D-AAs did not inhibit new bone formation in their ovine model after 4 months following local delivery of a D-Pro/D-Phe/D-Met mix. Finally, Sanchez Jr et al.^194^ investigated an equimolar mix of D-Met, D-Phe, D-Pro, and D-Trp within 5 or 10 wt% polyurethane scaffolds in a rat segmental femoral defect model and reported no adverse host responses. Although more work is needed, these studies highlight the promising nontoxic and potentially pro-osteogenic activity of some D-AAs.

## Discussion

**Fig. 6:**
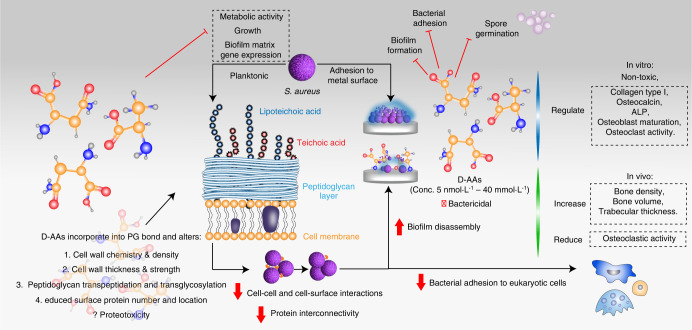
A schematic showing contemporary understanding of the role of exogenously applied D-AAs in regulating the bacterial species (e.g*., S. aureus*) associated with PJI in osteocompetent cells and bone tissue. D-AAs are not bactericidal but prevent pathogenic adhesion to surfaces and host cells, spore germination, and biofilm formation and are able to disassemble established biofilms. The mechanism(s) for this remains unknown. One route in which D-AAs may prevent bacterial adhesion is by reducing the number of hydrogen bonds (H-bonds) and thus adhesive forces to the surface. Furthermore, the mechanism of D-AA incorporation into the peptidoglycan bond may alter cell wall chemistry, density, thickness, and strength, thereby disrupting surface protein numbers and locations. This in turn may disrupt cell‒cell and cell-surface adhesion to abiotic surfaces and to eukaryotic host cells. The impairment of surface proteins may also decrease cell interconnectivity, thereby preventing biofilm assembly while also promoting biofilm disassembly. D-AAs have been shown to be nontoxic to host cells at lower concentrations that are able to regulate pathogenic activity. In terms of bone, D-AAs directly regulate collagen type I, osteocalcin, alkaline phosphatase (ALP), osteoblasts, and osteoclast activity in vitro. In vivo studies have reported a beneficial response to D-AAs in terms of bone volume, density, and architecture

With the number of TJAs continuing to rise, the current complications associated with antibiotic-resistant strains, as well as challenges in antibiotic development and research, makes it more important than ever to find novel approaches for the prevention and treatment of PJIs. Accumulating data indicate the pivotal role of D-AAs in regulating bacterial activity and biofilm integrity. Here, we show the diversity of D-AA activity, where to varying degrees, many were able to reduce bacterial cell adhesion to a surface and/or host cell, reduce the initiation of biofilm formation, and disassemble late-stage established biofilms. This was demonstrated in both gram-negative and gram-positive bacteria relevant to PJI and at concentrations as low as 5 nmol·L^−1^. Although there are few studies, D-AAs appeared cytotoxic to osteocompetent cells only at very high mmol·L^−1^ concentrations. Remarkably, the D-AA concentrations able to inhibit bacterial activity and biofilm function did not present parallel cytotoxicity to osteoblasts, BMSCs, osteoclasts, or fibroblasts;^195,197^ although, cytotoxicity to BMSCs at μmol·L^−1^ concentrations has been demonstrated.^319^ Together, these studies suggest that further investigation is warranted, and D-AA bioengineering has the potential to serve as an important future therapeutic strategy in the prevention and treatment of PJI. The mechanism/s of action largely remain unknown and are likely highly complex (Fig. 6). In brief, D-AAs are not bactericidal, and when administered exogenously, they reduce cell metabolic activity.^198^ Their misincorporation during protein synthesis may lead to proteotoxicity together with reduced expression of key biofilm matrix genes.^219^ Furthermore, D-AAs incorporate into peptidoglycan bonds, likely inhibiting protein binding to the cell wall, disrupting cell‒cell and cell-surface interactions via alterations in wall thickness, peptidoglycan transpeptidation, and transglycosylation, and reducing the number and localization of surface proteins.^117,190^ Together, this may influence protein interconnectivity between neighboring cells, inhibiting biofilm initiation as well as its structural integrity once established. Notably, in some species, D-AAs did not upregulate exopolysaccharide production or matrix operon activity. Furthermore, hydrophobicity or charge does not appear to explain the changes in bacterial activity.^171^ The data also mostly showed that equimolar mixtures of D-AAs were more potent, suggesting synergistic activity within the various D-AAs. The activities relevant to PJI involve the ability of D-AAs to regulate bacterial spore germination and the response of the host immune system to invading pathogenic bacteria; furthermore, D-AA affect bacterial adhesion to abiotic surfaces, disrupt of biofilm formation, and initiate biofilm disassembly. The D-AA interaction with *S. aureus*, the most common microorganism isolated with PJIs, was a topic more thoroughly investigated when compared to other bacterial species. However, molecules including D-Glu, D-His, D-Val, and D-Ser have not been characterized in this manner. This is of interest, as these D-AAs showed significant efficacy in studies involving gram-positive *S. mutans* and gram-negative *A. baumannii*. In contrast, D-AA-focused studies directed toward *S. epidermidis*, an avid producer of PJI biofilm, were very limited, and further study of the interaction of this species with D-AAs may be of significant value. Of note, D-Trp, D-Tyr, and D-Phe showed potential across a plethora of bacterial species associated with implant biofilms; however, many more D-AAs were effective against only one or more pathogens, and further work is needed to elucidate their roles. Biofilm-inhibitory and dispersive therapies will undoubtedly improve PJI healing outcomes. For example, potential future use could include D-AA coatings or a form of surface incorporation onto implant components applied either pre- or peri-surgery, which may not only aid in preventing early onset infection and biofilm formation but could also enhance adjacent bone repair and osseointegration. Similarly, the inclusion of D-AAs contained within resorbable bone substitute scaffold materials may offer the advantage of limiting infection and promoting bone regeneration within a large bone defect site. In the revision PJI surgical setting following delayed or late onset infection, it is conceivable that the use of D-AAs either within a gel or spray solution and applied directly onto the bone and soft tissue surface following debridement may aid in dispersing remaining biofilm within the wound site and in reducing the risk of reinfection and osteomyelitis. Furthermore, their use may not be limited to orthopedics, and future research for use in a clinical setting may prove worthy in dental and plastic surgery, as well as other fields that utilize implants. If successful, the use of D-AAs may in turn assist in reducing patient morbidity and health care costs, assuming that the infection can be managed with more ease following the implementation of these molecules*.* However, as D-AAs are not bactericidal, it is possible that they will be most effective as an adjuvant therapy to contemporary treatment with systemic antibiotics. Nevertheless, it is important to note that the progression of infection, the host cell response, and the characteristics of bacterial activity toward D-AAs may be different for PJIs in the human body compared with the PJI animal models presented in this review. Although clinical PJI isolates were used in some experiments in vitro, the potential beneficial impact of D-AAs in human PJI in vivo remains to be uncovered.

An additional and important potential future role for D-AAs in PJI is their emergence as molecular probes for live imaging. Hsu et al.^122,321,322^ introduced a family of novel fluorescent D-AAs (FD-AAs) designed with high specificity to covalently incorporate into peptidoglycan via endogenous bacterial transpeptidases, thereby fluorescently labeling and monitoring peptidoglycan. This has allowed visualization of the peptidoglycan on the surface of a cell, in situ and in diverse bacterial species in real time. Their broad application and biocompatibility have made FD-AAs an important and effective spatiotemporal tracking tool.^323^ Other recent approaches have taken advantage of key metabolic differences between prokaryotic and eukaryotic systems. Notably, Neumann et al.^324^ developed a D-AA radiotracer composed of D-[methyl-^11^C]methionine ([^11^C] D-Met) for positron emission tomography (PET) imaging and was able to selectively differentiate between active and sterile *E. coli* and *S. aureus* infections in vivo. More recently, the team developed a radiolabeled D-alanine (D-[3-^11^C]alanine) radiotracer and showed accumulation in both gram-negative and gram-positive pathogens. Prominent uptake of the radiotracer was observed and shown to be effective at detecting active infection in murine models of *S. aureus* discitis-osteomyelitis and *P. aeruginosa* pneumonia using PET imaging.^325^ Higher radiotracer activity was observed within joints with active infection compared to joints inoculated with sterile bacteria. Finally, Ogawa et al.^326^ found that D-AAs were more stable than their L-AA equivalents and developed a ^67^Ga-DOTA-(D-Asp)_n_ PET imaging agent with high accumulation in bone and rapid blood clearance in mice. Further studies are needed to examine the effectiveness of the D-Asp agent as a peptide linker for the targeted delivery of drugs to bone tissue. The use of FD-AAs and D-AA radiotracers in a clinical setting has not yet been explored. However, use of both as detectors of microbial presence during PJI surgery on the implant or tissue surfaces, as well as monitors of infection postsurgery, may accelerate diagnoses and treatment and significantly reduce the costs associated with PJI, both in terms of decreasing patient morbidity and within the health care system.

In conclusion, the synthesis and catabolism of D-AAs, the roles they play in bacterial physiology and structure, their ability to limit biofilm formation and augment disassembly, and their use as in situ and real-time in vivo microbial detectors and drug delivery agents are all areas that warrant further study. New insights may facilitate the development of new therapeutic and diagnostic strategies for diseases related to several systems within the body, as well as to bone and PJI.
